# Identification and Functions of lncRNAs in Fungi

**DOI:** 10.3390/ncrna11050072

**Published:** 2025-10-07

**Authors:** Javier Avalos, Adrián Perera-Bonaño, M. Carmen Limón

**Affiliations:** Departamento de Genética, Facultad de Biología, Universidad de Sevilla, Av. de la Reina Mercedes, 6, 41012 Sevilla, Spain; adrianpbona@us.es

**Keywords:** non-coding RNA, pervasive transcription, antisense RNA, intergenic ncRNAs, cryptic transcripts, epigenetic regulation, yeasts, dimorphic fungi, filamentous fungi

## Abstract

Long noncoding RNAs (lncRNAs) are transcripts generated by polymerase II, therefore subject to 5′ capping and 3′ polyadenylation, categorized as such when they are at least 200 nt in size and lack coding function. The lncRNAs were initially interpreted as spurious transcription products, but over the last two decades an increasing amount of evidence has accumulated for regulatory functions. They are found in all taxonomic groups, including bacteria, archaea, fungi, animals and plants. In fungi, global analyses anticipate their presence in higher numbers than initially expected considering the simplicity of these organisms. Except for the numerous studies performed in budding and fission yeast, relatively few lncRNAs have been investigated in sufficient detail in the rest of the fungi, but their number has increased steadily in recent years. The lncRNAs can be transcribed from intergenic regions or coincide totally or partially with protein-coding genes, in which case they are most frequently antisense transcripts. Their regulatory functions can be performed by a wide variety of mechanisms, both in *cis* on neighboring genes and in *trans* on distant genes or on proteins. Among the most frequent mechanisms are interference on the transcription of neighboring genes and generation of epigenetic modifications in the environment of target genes. Here, we review the most representative cases of global analyses of the presence of lncRNAs in fungal transcriptomes and describe the lncRNAs that have received more detailed attention.

## 1. Introduction

Long non-coding RNAs (lncRNAs) are a class of RNA transcripts that are larger than 200 nt in size and, unlike messenger RNA (mRNA), do not possess significant or recognizably functional reading frames, and therefore are not expected to encode proteins [1]. Initially they were considered functionless transcriptional products or mere “genetic noise”, but today it is known that many lncRNAs play important roles in regulating the expression of other genes through very diverse mechanisms, covering epigenetic, transcriptional and post-transcriptional levels [2,3]. The interaction of lncRNAs with their regulatory targets does not conform to easily predictable mechanisms. In many cases, these are non-canonical interactions, such as the formation of a triple helix with a double-strand DNA. The diversity of mechanisms makes them difficult to study, despite efforts to understand and classify them [4]. The synthesis of lncRNAs is carried out by the same process as that of mRNAs. That is, they are transcribed by RNA polymerase II and can undergo maturation steps at the level of intron splicing, and additions of 5′ cap and poly-A tail [5].

There are different types of lncRNAs. From the point of view of their location in the genome or their relationship with coding genes, they can be intergenic, or associated with coding genes, in which case they can be transcribed antisense to the coding sequence, be located in an intron, or even be variants of the coding strand [6]. In overall, lncRNAs may exert their action by interacting with other molecules in the cell [7], which may be DNA, other mRNAs, miRNAs, or proteins, ranging from transcription factors, chromatin, or proteins mediating different processes, and there are computational tools to predict many of these interactions [8]. When they are antisense, they can form double-stranded RNAs with their complementary RNAs, which can affect their stability and availability for translation [9,10,11,12]. In higher organisms, their expression is usually highly specific to the level of tissue, developmental stage or physiological conditions, suggesting specialized functions [13,14,15].

Discovery of lncRNAs arose from the development of global transcriptomic techniques, first with cDNA microarrays, followed by cDNA tiling arrays, and fostered in the last decade by the enormous development of high-throughput sequencing technologies, such as RNA-seq [5]. At present, the large amount of accumulated information makes lncRNAs a type of regulatory elements with a wide variety of functions and mechanisms that are increasingly better characterized, and for which there are well-established study procedures [16] and databases that keep growing, including many involved in human diseases [17]. Initiatives such as ENCODE [18] or NONCODE [19] revealed the existence of thousands of previously unannotated non-coding transcripts in the human genome or in other organisms. Their presence, however, is widespread in all species investigated. This review summarizes the current state of knowledge on lncRNAs in fungi, about which new information is currently accumulating exponentially.

## 2. Identification of Antisense RNAs and lncRNAs in Fungi

Global transcriptomic analyses, and more recently those based on RNA-seq technologies, have revolutionized the identification and study of RNAs of unknown functions and in all types of organisms, including fungi [20], with long non-coding RNAs (lncRNAs) as an outstanding class. Large-scale detection and quantification of any type of transcripts have revealed the wide diversity of lncRNAs involved in gene regulation of a wide range of processes [16]. The data obtained in fungi has expanded the knowledge about their transcriptional complexity and the evolution of their genomes and has allowed us to glimpse that lncRNAs play more important roles than might have been expected considering the apparent simplicity of these organisms.

Recent works are often focused on the identification of lncRNAs, as a functionally open group with similar transcriptional characteristics, but previous studies focused on non-coding RNAs, with special emphasis on antisense RNAs because of their obvious regulatory characteristics [21]. For the most part, the latter are in fact a subclass of lncRNAs.

### 2.1. Saccharomyces cerevisiae

**Figure 1:**
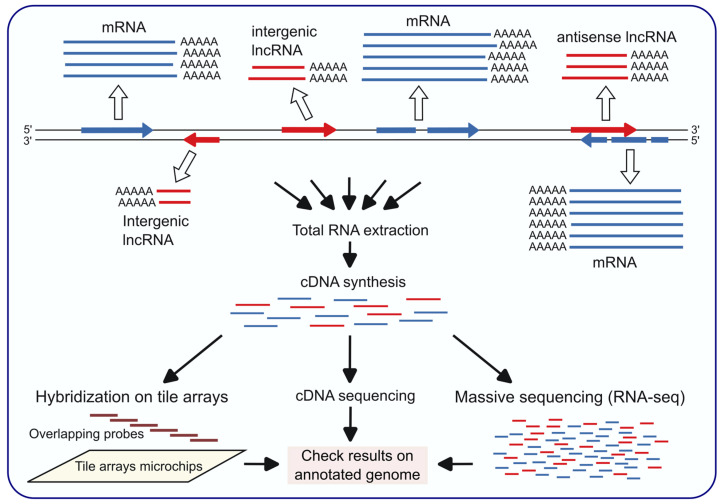
Procedures most frequently used for the detection of lncRNAs. Open and black arrows represent PolII transcription and experimental steps, respectively. Protein-coding sequences are indicated in blue. The two major classes of lncRNAs, antisense and intergenic, are represented in red.

The existence of transcripts that do not match annotated genes, or that match but are transcribed from the complementary strand (antisense transcripts), has been known in *S. cerevisiae* for more than two decades. Before RNA-seq techniques became available, the identification of non-coding transcripts of unknown functions, including the first lncRNAs, was initiated in *S. cerevisae* using tiling array technology [22,23]. This technique, consisting in the use of overlapping probes (tiles) that cover the entire genome (or specific regions of interest) in a uniform manner, without bias towards annotated genes, allows the detection of any type of transcripts, including non-coding and antisense transcripts (Figure 1).

In addition to genome tiling array, cDNA sequencing and RNA-seq analyses (Figure 1) of *S. cerevisiae* transcripts uncovered the existence of many non-coding RNAs [24]. The different techniques are complementary and give rise to different sets of lncRNAs, although they may overlap. For example, in some of the first studies the number of intergenic lncRNAs identified by cDNA analysis was 667 [25], by tiling arrays 234 [23], and by RNA-seq 487 [26]. Expression levels are highly variable, and in many cases are similar to those of coding genes. Overall, the transcription of non-coding RNAs is more extensive than expected, as indicated by the approximately 1000 intergenic or antisense transcripts detected by full-length cDNA analysis [25], subsequently corroborated by the identification of 1103 antisense transcripts by strand-specific RNA-seq analyses, many of them with differential expression depending on the growth conditions and conserved in other close species [27].

Not all antisense transcripts lack coding functions. The *S. cerevisiae MDF1* gene, a negative regulator of the mating pathway that interacts with MATa2, completely overlaps with the antisense gene *ADF1*, which encodes a protein that blocks *MDF1* transcription [28]. This is expected to be a rare case, due to coding constraints imposed by the overlap of the two sequences. On the other hand, the presence of an antisense RNA does not necessarily imply regulation of the involved protein-coding gene. In a study in which the transcription of antisense RNAs from 162 coding genes in *S. cerevisiae* was repressed and the impact on the amounts of proteins from their target genes was analyzed under four different culture conditions, a significant impact on protein levels in at least one condition was observed in only a quarter of them [29]. The effects were less likely for genes that showed high expression and more likely when the antisense RNA overlapped with the transcription start sites (TSSs).

The finding of lncRNAs in *S. cerevisiae* was promoted by global transcriptomics analysis of mutants affected in RNA degradation mechanisms. The reason for this is that, although non-coding lncRNAs undergo a maturation process at their 5′ and 3′ ends like that of coding mRNAs, many are not exported to the cytoplasm and remain in the nucleus performing short-term regulatory functions, until they are degraded by the exosome, an enzyme complex involved in RNA processing and degradation [30,31]. On the other hand, a population of more stable lncRNAs that are not rapidly degraded, described as Stable Unannotated Transcripts (SUTs), is also detected [32]. In both cases, they are frequently transcribed from nucleosome-free regions associated with the promoters of other genes or with their 3′ regions, from which bidirectional transcription is often observed [32,33]. The occurrence of unstable RNAs, initially called Cryptic Unstable Transcripts (CUTs), was evidenced in mutants affected in exosome function, where they accumulate abnormally [34]. The reason for their different behavior is that, unlike the reproducibility of the polyadenylation sites of the mRNAs to be translated, these lncRNAs have greater heterogeneity at their 3′ ends. In *S. cerevisiae*, this is due to their premature transcriptional termination by the Nrd1 complex, which interacts with the PolII complex and marks it for future exosomal degradation [35,36]. This mechanism plays an important role in the control of lncRNAs, as indicated by the 1526 transcripts that increase in the absence of Nrd1, which led to them being named NUTs, from Nrd1-unterminated transcripts [37].

As a representative example, transcriptome comparison between wild type and a mutant lacking Rrp1p, an essential component of the nuclear exosome, allowed the identification of 98 non-coding transcripts of 350–800 nt, half of them corresponding to promoter regions and the rest to intergenic or antisense transcripts [38]. On the other hand, comparison with the transcriptome of the mutant lacking Rrp6p, an essential component of the RNA processing exosome [39], revealed a strong increase in polyadenylated RNAs of sizes around 250–500 nt, largely associated with promoters [40]. Because of their length, they are not expected to be full-length mRNAs and they could regulate the transcription of downstream RNAs.

High-resolution oligonucleotide arrays were used to study the role of the nuclear exosome component Rrp6 in the transcriptome of *S. cerevisiae* involved in the vegetative and sexual phases of its life cycle [41]. The data allowed the identification of 1452 differentially expressed non-coding RNAs specific to meiosis, named as unannotated meiotic transcripts (MUTs). Rrp6 keeps MUT levels low during the budding stage, but the degradation of Rrp6 at the onset of meiosis leads to the accumulation of MUT in successive waves. As a result, diploid cells lacking Rrp6 cannot undergo premeiotic DNA replication and subsequent meiotic development. Therefore, Rpr6 plays a critical role in the asexual-sexual transition and the initiation of meiosis through differential ncRNA degradation depending on its activity levels.

The involvement of epigenetic regulation in the mechanisms of action of 566 antisense lncRNAs that increase in quantity in the absence of Rrp6 was also investigated using high-density tiling arrays [42]. For this goal, the regulation of the target genes was analyzed in mutants for the H3K4 histone methyl transferase Set1 or the histone deacetylases Hda1 and Rpd3. For 469 of the antisense lncRNAs, their increase in the Rrp6 mutant did not affect the transcription of their sense genes. Among the rest, for which the higher lncRNA level resulted in a repressing effect on the target gene, two types were found which differed in the silencing mechanism. For 69, silencing of the sense gene involved histone modification activities, as revealed by the effects of modifying enzymes, and for 28, silencing occurred through another mechanism, possibly transcription interference. The repressive effect of antisense transcription on expression of the sense genes was related to the efficiency of early termination of the antisense RNA and its polyadenylation. The data suggested that a subset of antisense lncRNAs, or their transcription, can recruit histone-modifying proteins to specific targets to control their expression.

A quality control checkpoint for aberrant transcripts, known as nonsense-mediated decay (NMD) pathway, is involved in the degradation of mRNAs with a premature termination codon, an upstream open reading frame, or an abnormally long 3′ UTR [43]. The transcripts recognized by the NMD machinery are degraded by exonuclease Xrn1 in the cytoplasm. In *S. cerevisiae* an important proportion of lncRNAs are targeted by NMD and degraded by Xrn1. These lncRNAs are known as XUTs (Xrn1-sensitive unstable transcripts), as their levels increase in the absence of Xrn1 activity [44]. The RNA-seq study with the mutant revealed 1658 XUTs, of which two-thirds are antisense to coding genes. XUTs are targeted to Xrn1 through the translation-dependent Nonsense-Mediated mRNA Decay (NMD) pathway [45], as indicated by their accumulation in mutants lacking Upf1, an RNA helicase essential for NMD, and Rrp6, a nuclear exosome catalytic subunit [46]. This yeast contains seven other cytoplasmic helicases, two of which have also been shown to be actively involved in the control of XUT levels [47].

Techniques based on the use of tile arrays or cDNA sequencing preceded those based on massive RNA sequencing. The latter, due to their affordability and high resolution, especially strand-specific sequencing, has gradually replaced other techniques, and has facilitated its extension to a growing number of fungi. Table 1 shows the results of the identification of antisense and intergenic lncRNAs in some representative cases of *S. cerevisiae* and the most informative ones of those presented in the following sections.

### 2.2. Other Yeasts

The content of antisense lncRNAs has also been analyzed in other species of budding yeasts, where they have been found in smaller numbers. Thus, while 2230 were detected in *S. cerevisiae*, their number was 810 in *Saccharomyces mikatae*, 525 in *Saccharomyces kudriavzevii*, and 431 in *Saccharomyces uvarum*, with very similar size distributions [61]. The lower number in other *Saccharomyces* species can be attributed to the partial loss of the RNAi machinery, totally absent in *S. cerevisiae*, since in a very closely related budding yeast species that retains fully active this machinery, *Naumovozyma castellii*, the number of antisense lncRNAs identified is only 177, and their average sizes are smaller. Although it has been proposed that the presence of cytoplasmic RNAi in *N. castellii* affects the antisense lncRNA transcriptome, in this species too, its levels are mainly controlled by the Xrn1 degradation system [62].

Another yeast for which lncRNA analyses have been carried out is *Schizosaccharomyces pombe*. A detailed study achieved in this yeast, combining RNA-seq and high-density tiling array techniques, and comparing different growth conditions, such as minimal or rich media, thermal, and oxidative stress, and different phases of meiosis, allowed for a highly detailed characterization of its transcriptome [48]. This work revealed the existence of transcripts for numerous previously unidentified genes, including 427 for lncRNAs, whose average sizes and expression levels were lower than those of coding genes. A high degree of bidirectional transcription throughout the genome was found, although in most cases transcripts from one of the strands predominate. Comparison of the data with those obtained with a *rrp6* mutant, affected in nuclear exosome function, showed that 36 of the new transcripts accumulate in greater quantities in the mutant, indicating that they are normally degraded in the nucleus. Later, a different protocol for the analysis of high-resolution tiling microarrays allowed the identification of 510 lncRNAs differentially expressed under oxidative stress [63].

A strand-specific RNA-seq study of the transcriptomes of *S. pombe* and three other related fission yeast species included a comprehensive survey of putative noncoding RNAs [49]. As a result, 1097 putative noncoding transcripts were found, of which 213 might be alternative UTRs due to their overlap with annotated UTRs on the same strand. Of the remaining 884 ncRNAs, 338 were intergenic and 546 antisense, most of them presumably lncRNAs. Of the 338 intergenic ones, 138 were conserved in location and 26 were conserved in sequence in at least another related species, suggesting the existence of potentially biologically important non-coding RNAs. Of the 546 antisense transcripts, 328 were conserved in two or species, also suggesting that they are functionally relevant.

A more exhaustive analysis of potential lncRNAs was carried out in *S. pombe* by comparing RNA-seq data from 12 mutants of nuclear and cytoplasmic RNA processing systems, and nine different physiological conditions, quiescent or stationary phase cells at different incubation times and several stages of meiotic differentiation development [64]. As a result, 5775 potential lncRNAs were identified, most of which had low expression levels but were induced in some of the conditions studied. They have been classified in three classes depending on the predominant system involved in their degradation, CUTs, degraded by the exosome, XUTs, degraded by Exo2/Xrn1, and DUTs (from Dicer-sensitive Unstable Transcripts), degraded by the RNAi machinery. The analysis was completed with their relationship with neighboring mRNAs and nucleosome positioning patterns, observing that many lncRNAs originate from nucleosome-depleted sites, often with bidirectional transcription. The three types of lncRNA degradation mechanisms have overlapping functions, as they can degrade the same lncRNAs, albeit with different affinities. Together they form a sophisticated RNA surveillance network that is partly responsible for the proper functioning of gene regulation in the cell to optimize its viability at different stages of its life cycle or under different growth conditions.

Other studies in *S. pombe* have also investigated different aspects of lncRNA accumulation. The histone chaperone Spt6 participates in the regulation of the expression of numerous genes by interacting with histones and RNA polymerase II. A combined approach in a *spt6* mutant, both at the transcriptome and chromatin structure levels, showed an increase in antisense RNAs in 70% of genes, probably due to alterations in histone modifications [65]. An RNA-seq variant, NET-RNA-seq, consisting in specific sequencing of nascent RNAPII RNAs, was applied to investigate antisense transcription in *S. pombe* and its connection with Exo2/Xrn1 through the effect of the *exo2* mutation [66]. This method, that detects only elongating transcripts, found antisense transcription in 3455 protein-coding genes (68% of total), mostly XUTS, thus increasing the catalog of antisense RNAs known to date in this yeast. A study on the role of histone deacetylation in the regulation of meiosis genes included a global analysis of CUTs, corresponding to those whose levels rise at least twice in a mutant lacking Rrp6, allowing the identification of some 2500 [67]. Noncoding RNA sets depend on the methodologies used. Improved genome annotation redefining mRNA borders allowed to detect 487 novel ncRNAs [68].

There are fewer examples of global lncRNA studies in other yeasts. An RNA-seq study of the methylotrophic species *Pichia pastoris* to investigate the effect of stress caused by overexpression of heterologous phospholipase A2 and exposure to methanol led to the identification of 208 lncRNAs, of which 168 were antisense, 36 intergenic, and 4 intronic [50]. As found in other yeasts, their average sizes and expression levels were lower than those of coding genes. Of the 208 lncRNAs, 18 showed differential changes in their expression in the investigated conditions, and three were chosen for a more detailed study, described in Section 4.3.

### 2.3. Dimorphic Fungi

Dimorphic fungi are characterized for their ability to alternate between yeast and filamentous forms in their life cycles. The ability for this developmental switch provides them enormous adaptability, allowing them to thrive in very different environments. This morphological plasticity, often linked to pathogenicity, makes them organisms with a great capacity to adapt to adverse situations or respond to environmental signals.

In its process of maize infection, the basiodimycete *U. maydis* changes from haploid yeast to dikaryon hyphae, which form thick-walled diploid teliospores that are used for dispersal [69]. In a sequencing study of expressed sequence tags (ESTs) libraries from different developmental stages, transcripts for 4675 genes were found, of which antisense transcripts were detected for 210 of them, most with possible ORFs but no real recognizable coding function [51]. Subsequent analysis of a cDNA library of dikaryotic cells brought the number of antisense transcripts to 247 and extended the analysis to the identification of more than a hundred non-coding RNAs, defined by the absence of ORFs with database matches [70]. Some of them could be associated with pathogenesis, according to their expression patterns (see Section 4.4.1).

Subsequently, a comparative study between *U. maydis* and two other species of the smut fungi group, *Ustilago hordeum* and *Sporisorium reilianum*, revealed a high number of lncRNAs, many shared and others species-specific [52]. Most of them were antisense or intergenic transcripts, with total numbers for the first class of 2624 in *U. maydis*, 1606 in *U. hordeum*, and 1949 in *S. reilianum*, about half conserved in all three species, indicating relevant regulatory functions. On the other hand, 2414, 1206 and 1776 intergenic transcripts were found, respectively, in the same species. Some of them could be protein-coding transcripts that escaped the annotation processes, but presumably the vast majority are true lncRNAs. The high numbers of lncRNAs found in these fungi reveal a high regulatory complexity in which lncRNAs play an important role.

In an RNA-seq analysis specifically aimed at identifying lncRNAs in the rice pathogen *Ustilaginoidea virens*, samples from five stages of infection were analyzed, accompanied by microscopic follow-up [53]. As a result, 1724 lncRNAs were found, of which 1084 corresponded to intergenic regions and 566 to antisense transcripts. Of the latter, some were antisense of transport genes, and one of them was investigated in detail (described in Section 4.4.4).

Due to its importance as a human pathogen, several studies focused on the role of lncRNAs in the pathogenic activity in *Candida* species. A comparative analysis of numerous transcriptomic datasets from five pathogens of this genus: *Candida albicans*, *Candida tropicalis*, *Candida parapsilosis*, *Candida auris* and *Candida glabrata* identified hundreds of lncRNAs, located in both intergenic regions and protein-coding genes [71]. Although many show low sequence conservation between species, some lncRNAs are syntenic and enriched in common motifs. Coexpression between certain lncRNAs and protein-coding genes was also detected, suggesting functional interactions. They also identified differentially expressed lncRNAs during infection of human epithelial cells in four of the species studied. The findings highlight the relevance of lncRNAs as potential regulators of virulence and adaptation in the genus *Candida*.

### 2.4. Filamentous Fungi

Filamentous fungi constitute the most extensive and heterogeneous group within fungi. Their multicellular growth form and the formation of hyphae, mycelia, and reproductive structures, the latter normally involved in the formation of asexual or sexual spores for dispersal, make many of them models of great interest for studies of development and differentiation. In many cases, they are pathogenic organisms, but they are also capable of living freely, for which they have developed highly versatile metabolisms. In some cases, they stand out for their ability to degrade biopolymers, such as cellulose or chitin, or to produce secondary metabolites with different biological properties, such as antibiotics or mycotoxins.

The ascomycete *Neurospora crassa* is an important model in molecular biology of filamentous fungi [72], with relevant contributions in the areas of photobiology and circadian rhythms, and with extensively developed genetic and molecular tools [73]. In this organism, which is non-pathogenic and does not produce mycotoxins, different illumination and temperature conditions were studied by RNA-seq and 939 lncRNAs were identified, evenly distributed across its seven chromosomes [54]. Of these, 477 were antisense with varying degrees of overlap with coding genes. Eleven lncRNAs were stimulated by light, opening up new fields in the photobiology of this fungus. In a subsequent study combining ChiP-seq, RNA-seq, and polysome fractionation, the number of lncRNAs rose to 1478 intergenic lncRNAs and 1056 antisense lncRNAs, most of which were shorter, typically without introns, and with lower expression levels than the average for coding genes [74]. This study highlights the importance of non-coding transcription in *N. crassa*, with ca. 20% of RNA polymerase II transcripts being lncRNAs.

*Aspergillus flavus* is a phytopathogenic fungus and opportunistic human pathogen of great interest due to its ability to produce mycotoxins, among which aflatoxin stands out for its health risks [75]. Several surveys have been carried out with this fungus. In an analysis of EST sequences by microarray technology, 352 antisense transcripts were detected [76]. Temperature is a determining environmental factor for aflatoxin production in infected maize. Temperature regulation was detected in 32 of these transcripts, some of them related to secondary metabolism and aflatoxin metabolism. A later RNA-seq analysis in mycelia and sclerotia in the same fungus showed that 30% of all transcripts were unknown, the vast majority, approximately one thousand, corresponding to antisense transcripts presumably involved in post-transcriptional regulation of coding genes [55]. The new ncRNA transcripts identified have globally similar expression levels to those of the coding genes, and 62% of these have lengths greater than 500 nt, so the vast majority must be lncRNAs.

Another RNA-seq study in *A. flavus* under different stress conditions, such as changes in water activity, CO_2_ concentration and temperature, identified 472 putative lncRNAs [77]. Many of them showed differential expressions to varying degrees under stress, suggesting key roles in aflatoxin biosynthesis, respiration, cell survival, and metabolism. In addition, some sense lncRNAs, downregulated by temperature, osmotic stress and CO_2_, could indirectly modulate proline metabolism. Subcellular localization analysis indicated that some regulated lncRNAs are concentrated in the nucleus, whereas others accumulate mainly in the cytoplasm. Possible regulatory targets based on chromosomal locations, correlation with the expression patterns of other genes, e.g., those for aflatoxin biosynthesis, and predicted interactions with milRNAs were suggested, laying the groundwork for further studies on specific regulatory mechanisms.

Numbers of lncRNAs were found to be very high in some fungi. Transcriptome analysis of *Cordyceps militaris*, an insect pathogenic fungus, identified 4140 putative lncRNAs, many of them manifesting changes in their transcript levels in the transition from mycelium to fruiting bodies [78]. As found in other fungi, on average, their sizes and numbers of exons were smaller than those of coding mRNAs, and a very high proportion of them are located at the 5′ region of neighboring genes, in many cases probably their regulatory targets. Deletion of the *xrn1* gene in *C. militaris*, with a central role in the NMD pathway [79] and affecting lncRNAs, reduced virulence on insects and slowed growth, strongly suggesting the involvement of lncRNAs in the pathogenesis and other aspects of the biology of this fungus.

A high number of lncRNAs was also found in the rice pathogen *Magnaporthe oryzae*. Transcriptomic analysis at 6 different stages of the infection process identified 2601 lncRNAs, including 1286 antisense and 980 intergenic lncRNAs [56]. A high proportion, 755, showed differential expression at different stages of infection and 560 were found specifically in pathogenesis. Many of them are neighbors of genes with pathogenesis-related functions or are unlinked but show complementarity with their sequences. It is concluded that regulation by lncRNAs in *M. oryzae* plays an important role in the regulation of genes required for pathogenesis.

An analysis aimed at completing the sequence and annotation of the genome of *Botrytis cinerea*, a pathogen of grapes, strawberry or tomato, and many other commercial plants [80] included a detailed preliminary analysis of the first of its 18 chromosomes, in which 30 antisense transcripts were found that had undergone intron splicing and that at least partially matched the coding sequence of the gene in which they are located [81]. A thorough survey of *B. cinerea* lncRNAs was recently carried out during different stages of infection on tomato, from inoculation to 48 h of growth in the plant [57]. As a result, 1837 lncRNAs were identified compared to a total of 18063 annotated coding genes, of which transcripts were detected for 14236. More than 40% of the lncRNAs possessed introns, but their average number, as well as the average transcript size, were lower than those of the coding genes. Of the 1837 lncRNAs, 743 were antisense, of which 55 were induced in the late stages of infection, in parallel to the overlapping coding genes. Interestingly, alternative splicing was observed in 123 of the lncRNAs, adding another level of complexity to their possible regulatory functions.

In the cotton pathogen *Verticillum dahliae*, the effect of nutrient starvation or growth on pectin as pathogenesis-associated conditions was investigated by RNA-seq in the wild-type strain and in five virulence gene mutants. As a result, 2965 putative lncRNAs were identified, distributed in similar numbers of intergenic and antisense lncRNAs [58]. Many of them were upregulated under starvation conditions or in the presence of pectin in different ways in the five mutants, with the highest numbers in nitrogen-deprived medium, indicating an association with pathogenesis. However, in another study with the same *V. dhaliae* strain, only 352 novel lncRNAs were detected [82]. In this case, RNA-seq was performed at three stages of infection on cotton, and 47 of the lncRNAs significantly changed their transcript levels during pathogenesis and parallel changes were detected in neighboring genes, presumably regulatory targets. In both studies, the involvement of some lncRNAs on pathogenesis was confirmed by the effects of their overexpression, as described in Section 4.5.10.

In an RNA-seq analysis of *Pyricularia oryzae*, a phytopathogen causing rice blast, 3374 previously unidentified genes were found, mostly putative lncRNAs, with a predominance of intergenic transcripts with parallel expression to neighboring genes [59]. Many of them changed their expression patterns in hyphae or conidia, suggesting that they may be involved in the regulation of development, a hypothesis that was confirmed for at least one lncRNA investigated in more detail (Section 4.5.8). As a last example on pathogenesis studies, genomic features of lncRNAs from the wheat pathogen *Zymoseptoria tritici* were identified by analysis of their expression during the infection cycle. Their characteristics and their distribution along the chromosomes were described as well as their differential expression during plant infection [83].

The species of the genus *Fusarium* are widespread plant pathogens, producing important losses in agriculture. *Fusarium graminearum* transcriptome data were obtained from five successive sexual stages and from hyphae to search for differentially expressed mRNAs and lncRNAs [60]. Among the 2574 noncoding transcripts, 547 were differentially expressed at least in one of the developmental stages. Of them, 280 were antisense lncRNAs that overlap at least 100 bp with an mRNA, 237 were long intergenic ncRNA (lincRNA). The expression of some lncRNAs were validated by amplification by PCR of their cDNA. Deletion of *xrn1* resulted in a defective sexual phenotype, although the mutant produced normal asexual conidia. Transcriptomics of wild type and Δ*xrn1* mutant from asci and vegetative stages were compared to search for lncRNAs involved in development of sexual fruiting body. Expression of 25 lncRNAs were upregulated in the Δ*xrn1* mutant in hyphae and increased in asci (meiotic stage).

### 2.5. Other Global Studies

The cases described above are representative of the works on fungi that are proliferating in increasing numbers based on computational analysis of transcriptomes. These are particularly powerful in the case of those that are strand-specific, as they allow the identification of antisense RNAs. Other examples on global lncRNA studies in fungi may derive from the study of the transcriptome in a single condition, as the analyses carried out in the human pathogens *Paracoccidioides brasiliensis* [84] and *Cryptococcus neoformans* [85]. However, the analyses usually compare different biological conditions and are aimed at associating lncRNAs with specific physiological processes, as those involved in regulatory effectors in the soy pathogen *Phytophthora sojae* [86], stress in the entomopathogen *Metharizium robertsii* [87], virulence decline in the aphid pathogen *Conidiobolus obscurus* [88], pathogenesis in the development in the pome fruit pathogen *Penicillium expansum* [89], heat-shock in the thermophilic fungus *Thermothelomyces thermophilus* [90], nucleus–mitochondria interaction in the edible fungus *Lentinula edodes* [91], growth rate in the biotechnological yeast *Komagataella phaffii* [92], or sexual and asexual development in the potato pathogen *Phytophthora infestans* [93].

## 3. Mechanisms of Action of Fungal lncRNAs

In recent years, fungal lncRNAs have been reviewed, including their proposed mechanisms of action. The reviews are either general [94,95] or specifically focused on yeasts [96,97,98] or antisense lncRNAs [99]. The cases of lncRNAs studied in more detail allow us to sketch a general scenario of the different regulatory mechanisms in which they may be involved. This section may serve as a guide for a better understanding of possible mechanisms for the specific lncRNAs described in Section 4, especially for those in which there is sufficient experimental support.

**Figure 2:**
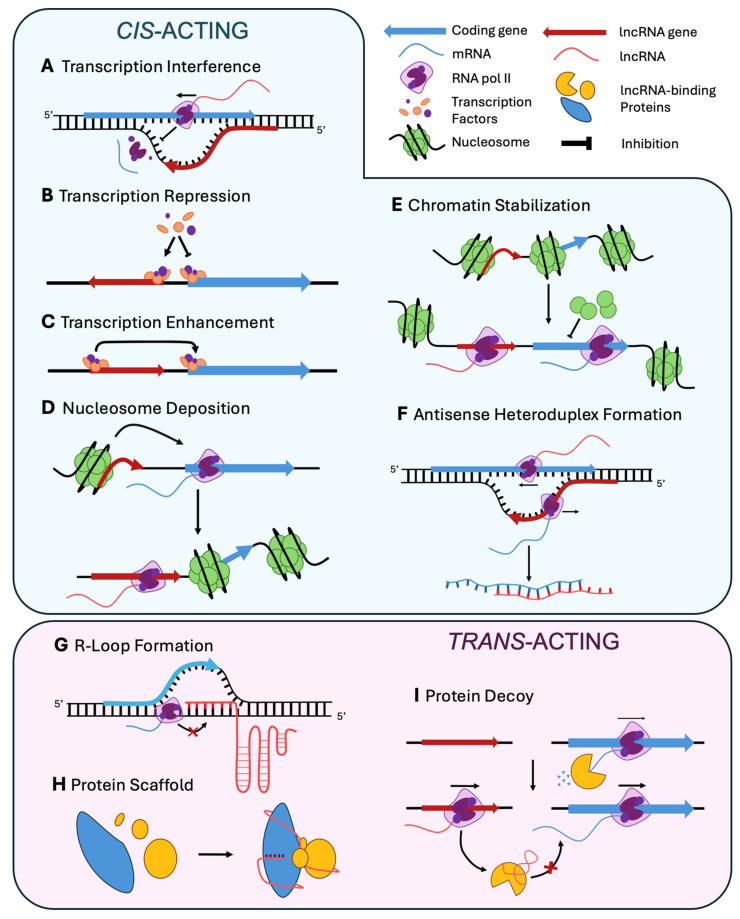
Representative examples of the mechanism of action of lncRNAs in fungi. (**A**–**F**) LncRNAs acting in *cis*. (**G**–**I**) LncRNAs acting in *trans*. Arrows crossed out in red indicate inhibitory effects.

As a first criterion for classification, there are *cis*- and *trans*-acting lncRNAs. *Cis*-acting lncRNAs affect immediately close regions of the genome, and they can be in sense or in anti-sense orientation with respect to the region they regulate. The regulatory effects of some lncRNAs are a consequence of their own transcription, being able to negatively affect the transcription of genes in the opposite or same strand. This ‘Transcriptional Interference’ (Figure 2A) can be caused by transcriptional machinery collision, promoter occlusion, or interferences with the initiation or elongation of overlapping or nearby genes (e.g., *RME2* on *IME4* in *S. cerevisiae*, ref. [100]). In other cases, a ‘Transcription Repression’ (Figure 2B) may occur via histone modification associated with the ability of lncRNA to modify complexes such as histone deacetylases and histone methyltransferases (HMTs), altering chromatin accessibility and reducing transcriptional activity. An example is provided by *PHO84* antisense on *PHO84* in *S. cerevisiae* [101,102]. However, not all lncRNAs act as inhibitors, and their action may result in ‘Transcriptional Enhancement’ (Figure 2C), as occurs with *PHO5* antisense on *PHO5* in the same yeast [102,103]. In these cases, lncRNAs facilitate activation of neighboring genes by promoting chromatin accessibility, typically recruiting histone acetyltransferases (HATs) or transcription factors. The transcription of lncRNAs can strongly alter the nucleosome environment resulting in ‘Nucleosome Deposition’ (Figure 2D) over adjacent promoter regions, thereby repressing gene expression, as described for *SRG1* on *SER3* in *S. cerevisiae* [104,105,106]. This mechanism is prominent in tightly packed genomes and can serve as a rapid response to developmental or environmental signals. Other lncRNAs act as chromatin stabilizers by maintaining either active or repressed chromatin states. This ‘Chromatin Stabilization’ (Figure 2E) mechanism can involve interactions with chromatin readers or remodelers that prevent the spread of heterochromatin or protect euchromatin from silencing. A good example is the action of *Ty1* antisense on Ty1 transposon in *S. cerevisiae* [107]. Such stabilization is frequently observed in pericentromeric or sub-telomeric regions, where the chromatin landscape is dynamic.

LncRNAs can interact with other functional molecules, either RNA, DNA or proteins. LncRNA-mRNA interaction involves ‘Antisense Heteroduplex Formation’ (Figure 2F), as found with *as-ssm1* on *ssm1* in *U. maydis* [108]. In this mechanism, antisense lncRNAs base-pair with complementary mRNAs to alter their stability or translation, protect them from degradation or induce granules formation. Interaction with DNA or proteins can expand the location of action of lncRNA, which shows a wide spectrum of effects. A characteristic lncRNA-DNA binding mechanism is the ‘R-loop Formation’ (Figure 2G), in which lncRNAs directly interact with genomic DNA through sequence complementarity or structural motifs. These interactions can promote the formation of lncRNA: DNA hybrids creating R-loops, which influence local chromatin accessibility, transcription, or recombination. As a paradigmatic case, lncRNAs derived from telomeric regions, such as TERRA of *S. cerevisiae* [109], bind DNA to modulate telomere length or genome stability. Such mechanisms may also be behind the long-range regulation of target genes by enhancer-associated lncRNAs that fold into tertiary structures capable of bridging distal genomic elements. LncRNAs may also work as ‘Protein Scaffold’ (Figure 2H) that facilitate the assembly of protein complexes by bringing together multiple components with distinct binding domains. A representative example is provided by the action of *meiRNA* at *sme2* locus in *S. pombe* [110,111]. This mechanism is particularly effective in the coordination of chromatin modifiers, splicing factors, or signaling proteins. Such scaffold functions are highly sensitive to lncRNA structure, expression levels, and cellular localization, often allowing for fine-tuned regulatory control across multiple processes. Other lncRNAs operate as ‘Protein Decoy’ (Figure 2I), binding to regulatory proteins and preventing them from interacting with functional targets, as proposed for *HAX1* on the Xyr1 activator in *Trichoderma reesei* [112]. This competitive inhibition mechanism can modulate diverse regulators, as transcription factors, kinases, or RNA-binding proteins.

## 4. Functions of Specific lncRNAs Investigated in Fungi

The study of long non-coding RNAs (lncRNAs) in fungi has revealed important roles for these transcripts in gene regulation, cell development, and response to environmental conditions. This section is a recompilation of the studies on specific fungal lncRNAs. The diversity of the cases studied provides insight into the heterogeneity of the processes and mechanisms of action in which they are involved, which are summarized in Figure 1. Special attention is paid to the yeasts *S. cerevisiae* and *S. pombe*, which stand out for the magnitude of research carried out.

### 4.1. Saccharomyces cerevisiae

The finding of pervasive transcription as a widespread phenomenon in *S. cerevisiae* suggested a high transcriptional complexity in this simple eukaryote [113,114], indicating the existence of additional levels of regulation beyond those previously known [115]. The functions of numerous lncRNAs have been investigated in more detail in this yeast. They are listed in Table 2.

#### 4.1.1. Metabolism and Cellular Functions

A classic example in budding yeast, and one of the best known at the mechanism level, is the lncRNA *SRG1*, which is transcribed upstream of *SER3*, a structural gene of the serine biosynthetic pathway, and represses its transcription [106]. This repression is regulated by serine via the activator Cha4 and coactivators SAGA and SWI/SNF. Additionally, *SRG1* transcription directs nucleosome deposition over the *SER3* promoter, blocking transcription factor access [104,105]. This process depends on elongation factors Spt6 and Spt16, which maintain nucleosome positioning without affecting *SRG1* transcription levels. Furthermore, Spt2, an RNA polymerase II-associated factor, facilitates nucleosome reassembly behind transcribing RNAP II, reinforcing repression [143].

Two genes involved in nucleotide metabolism, *URA2* in pyrimidine synthesis and *IMD2* in inosine synthesis, share a similar regulatory mechanism mediated by TSS selection [116,117]. Both genes are only induced when there is a shortage of a base involved in their pathway, uracil in the first case and guanine in the second. Regulation is carried out by the use under repressive conditions of an upstream TSS, which results in the synthesis of a SUT that is rapidly degraded. Under induction conditions, a TSS closer to the start codon is used instead, which gives rise to a stable and functional mRNA. In the case of *IMD2*, SUT transcripts (called here “attenuated transcripts”) start with guanine, which facilitates their preferential transcription in the presence of abundant GTP.

One of the lncRNAs identified in the transcriptomic analysis of the mutant lacking the exosome component Rrp1p was *HRA1* (from hidden in reading-frame antisense), antisense of the *DRS2* gene, which encodes a protein associated with the Golgi apparatus [38]. However, a role in the maturation of 18S rRNA was attributed to this gene because of its mutant phenotype. The authors suspect that this second phenotype is due to the loss of *HRA1* and not to the loss of the *DRS2* gene itself, providing an example of possible multifunctionality of the same DNA sequence.

A well-studied example of regulation by lncRNA in *S. cerevisiae* is the *GAL1-10* gene regulon [118], which responds to sugar availability, and which exhibits unusual Set1-dependent methylation at *GAL10* (H3K4me3) under repressive conditions [119]. This repression is associated with the transcription of the *GAL10*-ncRNA, transcribed in the opposite direction to *GAL10* at a very low rate, approximately once every 50 min. Its transcription depends on the binding of the Reb1 protein to specific sites and is rapidly degraded by the TRAMP and exosome complexes, keeping it at very low levels (∼1 molecule/14 cells). H3 methylation delays the recruitment of RNA polymerase II (RNAPII) and TBP on *GAL1* promoter. In addition, transcription of *GAL10-ncRNA* recruits Set2 and induces histone deacetylation through the Rpd3S complex, modifying chromatin structure. Other genes of the *GAL* regulon are also associated with lncRNAs, whose levels have been shown to be regulated by 5′ decapping, which results in their destabilization [144]. The *GAL* genes, as well as other inducible genes in *S. cerevisiae*, have evolved the use of regulatory lncRNAs to maintain their inactive state through an epigenetic mechanism, which involves histone modifications. Their regulation by decapping is evidenced by mutants of the enzyme that carries out this process, DCP2, which show increased expression levels of the *GAL1*, *GAL2*, *GAL4*, and *GAL10* genes.

Two lncRNAs involved in the regulation of phosphorus utilization have been analyzed. In the absence of Rrp6, the expression of the *PHO84* gene, which encodes a phosphate transporter, is repressed. This led to the detection of a regulation of *PHO84* by two antisense transcripts, whose levels remain higher in the absence of Rrp6 and repress *PHO84* transcription [103]. Under these conditions, there is a recruitment of the histone deacetylase Hda1 to the *PHO84* region, indicating that regulation by these antisense RNAs takes place at the epigenetic level, promoting deacetylation of histones by the Hda1/2/3 complex. On the other hand, the *PHO5* gene, which encodes an acid phosphatase that is excreted into the medium to hydrolyze organic phosphates, is regulated by another 2.4 kb antisense RNA, which is transcribed from the *PHO5* terminator and covers the entire gene [101]. In the wild-type strain, antisense transcript levels are low in the absence of P and higher in its presence or in mutants lacking Rrp6, and *PHO5* mRNA levels show the opposite pattern. However, loss of this transcript in a mutant lacking the 3′ region of *PHO5* results in a delay in chromatin remodeling, and subsequent recruitment of polymerase II. Thus, transcription of this antisense transcript promotes *PHO5* activation, possibly facilitating proper nucleosome positioning. A search for new factors participating in antisense RNA-mediated transcription interference led to the identification of the HIR histone chaperone complex, involved in histone deposition on DNA in replication-independent chromatin assembly [145]. Antisense RNA repression of genes whose expression is mediated by the SAGA complex requires HIR activity, which was also demonstrated for the *PHO84* and *PHO5* genes [102]. Knowledge of chromatin dynamics in the *PHO5* promoter, in parallel with the coregulated *PHO84* promoter among others, constitutes a paradigmatic model of epigenetic regulation of eukaryotic promoters [146].

A very different regulatory system has been described for the *ASP3* gene, which encodes an asparaginase that is activated only in the absence of nitrogen in the medium. An important role in its activation is played by an RNA transcribed from the central region of the gene in the sense orientation [120]. This unusual transcription occurs irrespective of the nitrogen availability and is independent of the *ASP3* GATA activators Gat1p and Gln3p, and of polymerase II itself, but results in an appropriate level of trimethylation of histone H3 at lysine 4 (H3K4me3) at the *ASP3* promoter in a manner that facilitates gene activation in the absence of nitrogen. This is therefore a case of positive epigenetic regulation by an internal sense transcript.

Target of Rapamycin (TOR) is a protein kinase that acts as a central nutrient and energy sensor, regulating cell growth and metabolism in response to environmental signals [147]. Its regulatory targets include amino acid transporters, and different lncRNAs found near transporter genes are assumed to be involved in their regulation. This is the case of the lncRNA *XUT_2F-154* (*TBRT*), antisense to the transporter gene *TAT1*, which regulates this gene and the linked transporter gene *BAP2*. The expression of all three is TOR-dependent, and when TOR activity is inhibited, *TBRT* levels fall and *BAP2* and *TAT1* mRNAs increase, and the same result is obtained by blocking *TBRT* transcription [121]. It is concluded that TOR keeps the expression of these two transporters under control by inhibiting them through *TBRT*, which takes place at the epigenetic level, establishing a state of repression in chromatin structure.

The *ATP16* gene encodes a subunit of mitochondrial ATP synthetase, which plays a key role in energy generation by coupling proton translocation to ATP synthesis. Upstream of *ATP16* there is a lncRNA gene called *CUT60*, whose transcription facilitates *ATP16* transcription [122]. For this purpose, it is necessary that *CUT60* terminates transcription at the appropriate site to facilitate accessibility of the transcription machinery to the *ATP16* promoter, since if the termination signal is removed and transcription of *CUT60* is prolonged, *ATP16* expression is greatly reduced, resulting in loss of mitochondrial function and a *petite* phenotype.

In an investigation of biotechnological interest due to the use of *S. cerevisiae* for the production of recombinant enzymes, the effect of the deletion of 208 intergenic lncRNAs, chosen on the basis of being at least 200 bases from the start codon of the nearest gene, on the secretion of α-amylase from an engineered plasmid was analyzed [123]. As a result, 21 lncRNAs were found to influence production, and of these, the deletion of *SUT067*, *SUT433*, and *CUT782* allowed for the greatest increases. The three deletion strains showed enhanced energy metabolism and translational activity, allowing for a greater supply of ATP and protein synthesis. This study shows the applied interest that lncRNA-mediated regulatory mechanisms may have.

Another extensive study investigated the phenotypic consequences of 372 mutants with deletions for ncRNAs, which included 50 CUTs and 93 SUTs [124]. Their phenotypes were tested under 23 different environmental conditions, mainly changes in carbon sources and stress conditions. As a result, 29% of SUTs and 22% of CUTs exhibited phenotypic alterations. Of these, 19 were selected, together with a snoRNA (small nucleolar RNA), to analyze the consequences of their deletions on the transcriptome. Six of them showed changes in the expression of hundreds of genes, while others showed more limited effects. Of the first, the deletions of SUT125, SUT126, SUT035, and SUT532 showed the most severe changes in the greatest number of conditions and were investigated in more detail. The number and diversity of the affected genes indicated an action of these ncRNAs on transcription factors, leading to cascading effects.

#### 4.1.2. Cell Cycle and Stress Responses

One strategy to identify lncRNAs potentially related to cell cycle control is to detect those that oscillate throughout the cycle. This approach revealed that 80 of 523 antisense transcripts undergo cyclical changes in accordance with the cell cycle or are antisense to genes that do so [125]. On the other hand, of 135 intergenic RNAs, 11 show cyclical changes in their levels, giving a total of 91 non-coding RNAs potentially associated with cell cycle regulation. These include antisense RNAs for well-known genes such as *FAR1*, *CTF4*, and *TAF2*. *FAR1* plays a key role in the G1/S transition, and it is expressed at the M/G1 transition and inactivated in the late G1 phase. Its antisense RNA shows the opposite pattern, as it appears when *FAR1* mRNA disappears. *CTF4* is required for the maintenance of chromatin structure to overcome the S-phase checkpoint, and its mRNA peaks at the G1/S and G2/M transitions, whereas its antisense RNA peaks at the G1/S and G2/M transitions. Other genes are not directly related to the cell cycle, but their regulation is connected to it. This is the case of *TAF2*, which assists TFIID in the initiation of transcription, and indirectly participates in cell cycle regulation by ensuring the expression of genes necessary for cell growth and division. The *TAF2* gene shows peaks of expression in late M phase and early G1, while its antisense RNA peaks at the end of G1 and throughout S phase.

A relevant case of cell cycle gene regulated by lncRNA is *CDC28*, which encodes cyclin-dependent kinase 1. One of the transcription factors regulating this gene is Hog1, the protein kinase that executes the response of the osmotic stress signal transduction chain. This response consists of the activation of transcription of hundreds of stress-responsive genes to adapt to the new conditions [148]. An analysis by whole-genome tiling arrays revealed that Hog1 also activates approximately one hundred lncRNAs, one of them an antisense lncRNA of the *cdc28* gene, also regulated by Hog1 [127]. The CDC28 kinase plays a key role in the control of the cell cycle, which is temporarily stopped in response to stress pending its resumption once adaptation has occurred. In this case, the transcription of this lncRNA exerts a positive effect on *CDC28* transcription, an action it exerts by facilitating the correct positioning of Hog1 and the Chromatin Remodeling Complex to facilitate the initiation of *CDC28* transcription and thus the resumption of the cell cycle.

When *S. cerevisiae* cells are under severe nutrient stress, they enter a resting stage known as quiescence. In a global analysis of antisense RNAs, comparing the wild-type strain and an NMD *upfl1* mutant, it was observed that many genes expressed in quiescent cells show very low expression levels in the exponential phase, and that this repression is often associated with the presence of antisense RNAs, very often also covering the promoters. This cause-effect relationship was confirmed for the *PET10*, *CLD1*, *MOH1*, and *SSH3* genes by prematurely interrupting the expression of antisense RNAs [126].

Ethanol stress is an important environmental scenario in *S. cerevisiae*. Among the hundreds of lncRNAs in *S. cerevisiae*, those that change their levels under high ethanol concentrations have been identified, and considerable differences have been observed between strains with different tolerance [149,150]. The ethanol stress response is complex, affecting numerous aspects of cell metabolism and function. In a strain with low ethanol tolerance, the lncRNA *lnc9136* has been studied by computational analysis and the effect of its deletion [128]. The results led to the conclusion that *lnc9136* acts by preventing ethanol-induced mitosis arrest; this is done by facilitating the binding of the cell cycle regulatory proteins Swe1p and Clb1/2 to the proteins Gin4p and Hsl1p. On the other hand, computational predictions of lncRNA *lnc10883* in a highly ethanol-tolerant strain indicated that it bypasses DNA damage and the mitotic spindle checkpoints to allow cell cycle progression through its interaction with Mec1p, involved in detecting DNA damage, or Bub1, involved in checking the correct attachment of chromosomes to the mitotic spindle. Analysis of RNA and protein levels under stress conditions with respect to control cells led to the conclusion that *lnc9136* also acts on the general level of translation in the cell [151], which is attributed to its action on the levels of 17S rRNA and Rrp1, a nucleolar protein involved in the processing of rRNAs [152]. On the other hand, the same experimental approach suggested that *lnc10027*, from a different strain, inhibits the formation of processing body and stimulates translation activity, linking ethanol stress to these cellular processes.

#### 4.1.3. Sexual Cycle and Development

At least two lncRNAs play a relevant role in the control of meiosis entry in *S. cerevisiae* [129,131]. The gene *IME1* (Inducer of Meiosis 1) is downregulated by the lncRNA *IRT1* (IME1 Regulating Transcript 1). *IRT1* is transcribed upstream towards *IME1*, and its transcription inhibits *IME1* expression through competition with the *IME1* promoter. In addition, *IRT1* acts by remodeling chromatin, as its transcriptional elongation recruits histone deacetylases that generate a closed chromatin state, such as Rpd3, blocking the binding of *IME1* activators. Moreover, its transcription induces repressive marks, such as H3K36me3, that maintain the region in a non-permissive state. Expression of *IRT1*, as that of *IME1*, is regulated by nutrient availability through the PKA and TORC1 activities [130]. Under conditions of sufficient nutrients, *IRT1* is repressed, but this repression is lost in the absence of PKA and TORC1. In turn, *IRT1* is controlled by another lncRNA located upstream, *IRT2*, transcribed in the same orientation. In haploid cells, *IRT2* has a low level of expression, which facilitates acetylation in histones H3 in the *IRT1* promoter region, and therefore its transcription, resulting in low expression of *IME1* [132]. However, in diploid cells, *IRT2* is actively expressed, preventing such acetylation and leading to low levels of *IRT1* transcription and therefore the derepression of *IME1*, enabling entry into meiosis.

Another gene required to initiate meiosis, *IME4*, is also regulated by an antisense RNA [100]. In haploid cells, *IME4* antisense RNA is produced, and in diploid cells, *IME4* mRNA is produced instead. The system appears to be regulated by interference between the transcription of both transcripts. In diploid cells, the heterodimer formed by transcription factors a1 and α2, encoded by the *MATa* and *MATα* loci of each sex, prevents transcription of the antisense *IME4* gene by binding to a site near its 5′ region, thus facilitating expression of the sense *IME4* and the ability of the cells to enter meiosis under the right environmental circumstances. Another case of regulation by antisense RNA of a gene activated by the a1/α2 heterodimer is *ZIP2*, involved in the process of recombination between homologous chromosomes in meiosis. This gene is negatively regulated by the lncRNA *RME3* [133]. In diploid cells, this antisense transcript is blocked by a1/α2, thus facilitating the transcription of *ZIP2*. These same authors named *RME1* and *RME2*, from Regulator of Meiosis, the antisense lncRNAs that inhibit *IME1* and *IME4* in haploid cells.

The expression of the *FLO11* gene produces a morphological alteration in yeast growth, leading to the formation of pseudohyphal filaments [153]. This gene has two epigenetically controlled states, induced or repressed. In the same clone of cells incubated under the same conditions, the cells show one or the other state, giving rise to a variegated phenotype. The alternation between the two states is controlled by the simultaneous action of two lncRNAs, *PWR1* and *ICR1*, which are transcribed in opposite and overlapping directions in the upstream region of the *FLO11* gene [134]. Transcription of *ICR1* results in chromatin compaction of the *FLO11* region, preventing its expression, whereas transcription of *PWR1* prevents *ICR1* transcription and thus promotes *FLO11* expression. Consequently, *FLO11* expression depends on whether *PWR1* or *ICR1* is expressed predominantly, which in turn depends on competition between an activator of *PWR1* transcription, Flo8, or a repressor, Sfl1. In some cells the balance is inclined towards *PWR1* and *FLO11* is inhibited (yeast-like phenotype), and in others it is inclined towards *ICR1* and *FLO11* is activated (filamentous growth), giving rise to the variegated appearance of the colony.

The cell wall is an essential structure for fungal survival and development, and its correct maintenance is subject to a fine control of the numerous genes involved, in which different lncRNAs participate in *S. cerevisiae*. In addition to *FLO11*, involved in this process, lncRNAs have been found controlling the *ECM3* [136], *PIR3* [137], *SPS100* [138] and *TIR1* [44] genes, regulated respectively by the lncRNAs *EUC1* and *SUT228*, transcribed from the same strand upstream of their target genes, and the antisense lncRNAs *SUT169* and *TIR1axut* [137]. In some cases the regulation is positive and in others negative. Many more cases are expected, since of 201 cell wall-related genes, overlap with antisense RNA is detected in 88 and overlap of their promoters with sense RNA in 15, most of which are likely to be functional regulatory lncRNAs.

The *HO* gene encodes an endonuclease involved in the switching of the mating type by a gene conversion mechanism [154]. Regulation of the *HO* gene is coordinated with the cell cycle, as it is always repressed except at the end of G1 phase, at which time it is induced following sequential binding of the SBF complex [155]. Cells can be stalled in G1 phase by nutrient starvation or pheromone exposure. If the cycle resumes due to the availability of nutrients, the persistence of SBF and Mediator complexes bound to the promoter causes the *HO* gene to be expressed again in the G1 phase of the next cell cycle. However, if such resumption is due to the disappearance of pheromone signals, *HO* remains repressed in the next cycle due to the action of a lncRNA, called *pHO-lncRNA* [139], which is transcribed 2700 nt upstream of *HO* and displaces the SBF complex. In that case *HO* is not expressed again until the G1 phase of the second cycle. In congruence with this mechanism, *pHO-lncRNA* only has an effect in *cis*, and its reintroduction in *trans* in a strain in which *pHO-lncRNA* transcription is blocked in *cis* does not restore wild-type regulation.

Genes whose expression shows a high variability or responds to the presence of a certain signal frequently overlap with antisense RNAs, suggesting their participation in its regulation. This was demonstrated for the *SUR7* gene, which is largely expressed in media containing galactose and is repressed following stimulation with the α-factor pheromone, while its antisense, *SUT719*, is expressed equally in both conditions [135]. The elimination of *SUT719* expression leads to derepression of the *SUR7* gene in the presence of the α-factor. The result indicated that *SUT719* expression leads to threshold-dependent regulation of *SUR7* expression, specifically inhibiting it when induced at low levels.

#### 4.1.4. Genome Integrity

The *S. cerevisiae* genome contains Ty transposable elements [156]. One of them is the Ty1 retrotransposon, whose activity is controlled by an antisense RNA, produced by polymerase II and covering from the middle of one of the transposon genes to the 5′ LTR [107]. Its mode of action consists of silencing gene expression by promoting histone deacetylation and methylation in the transposon region. The silencing activity of this antisense RNA is attenuated by its destabilization by the 5′ RNA degradation pathway through its exoribonuclease Xrn1. This is therefore an epigenetic regulatory mechanism in which the possible hybridization between sense and antisense RNA plays no role.

Sets of lncRNAs transcribed from centromeric regions play an important role in kinetochore formation [157,158], which is necessary for proper chromosome segregation in cell divisions. The centromeres of *S. cerevisae* are very short, about 125 bp and lncRNAs transcribed from the centromeres, called *cenRNAs*, are polyadenylated and have a longer size [140]. Such transcription is induced in the S phase of the cell cycle, is dependent on centromere-specific binding of CENP-A, a histone H3 variant, and replication, and is inhibited by the kinetechore protein Cbf1 and a variant of histone H2A. The amount of *cenRNAs* is finely regulated through the balance between their transcription and their degradation by the nuclear RNA decay pathway and is important for proper chromosomal stability and segregation [159].

Two lncRNAs play important cellular functions through their roles in telomerase activity and maintenance [160], the telomerase RNA *TLC1*, and the telomeric repeat-containing RNA *TERRA*. *TLC1*, known as *TER1* in *S. pombe* [161,162], is used as a template for reverse transcription to synthesize telomeric DNA and also acts as a flexible scaffold for the telomerase and as a tether for holoenzyme protein subunits [141]. On the other hand, *TERRA* is transcribed from subtelomeric regions towards the telomeres and includes subtelomeric sequences at the 5′ segments [109]. *TERRA* length varies due to the different TSSs for RNA polymerase II and the processing of the 3′ end, which at least in some cases are polyadenylated. *TERRA* is transcribed from the C-rich strand, so the RNA itself is G-rich, allowing it to hybridize to the telomerase RNA template and inhibit telomerase activity in vitro. Changes in *TERRA* expression can cause functional alterations in telomeres, altering their length, and interfering with replicative machinery. *TERRA* co-localizes in vivo with telomerase and can form RNA:DNA hybrid structures at telomeric ends called R-loops, which have been linked to the onset of the initiation of senescence and to an alternative form of telomere elongation [163]. *TERRA* is conserved from yeast to humans, where its role as a telomere-associated lncRNA implicates it in telomere length regulation and in diseases such as cancer [164].

A synthetic genetic array procedure, which allows to assign RNAs to specific cellular processes, led to the identification of *SUT457* as a lncRNA involved in telomere organization [142]. This was confirmed by studying the effect of its deletion, which accelerated senescence in telomerase deficient cells and led to the accumulation of single stranded telomeric DNA, an effect that requires Exo1 function. Ectopic expression of *SUT457* in the deletion strain suppressed telomeric overhang accumulation, indicating a *trans*-acting role for this lncRNA in telomere overhang homeostasis.

### 4.2. Schizosaccaromyces pombe

Like *S. cerevisiae*, *S. pombe* is a very simple and amenable eukaryotic model microorganism with a compact genome, widely used in research, and in recent years also in lncRNA studies. LncRNAs investigated to date in this yeast (Table 3) are described in this section.

#### 4.2.1. Metabolism, Cellular Functions and Stress Responses

The expression of the *fbp1* gene, encoding fructose-1,6-bisphosphatase, is a well-known model for the relationship between chromatin structure and transcription factor accessibility, uncovering a multilevel regulatory process in which lncRNAs play an important role [176]. Transcription of *fbp1* is strongly increased under glucose starvation, and such activation is facilitated by chromatin remodeling. This is produced by histone acetylation [177] triggered by the successive transcription of three lncRNAs, *mlonRNA-a*, *mlonRNA-b*, and *mlonRNA-c* [165]. Consequently, the insertion of a transcription terminator upstream of the regulatory region prevents the transcriptional cascade of lncRNAs and thus the opening of chromatin. On the other hand, transcription of these lncRNAs, which overlap upstream of the promoter and coding region of *fbp1*, promote in the absence of glucose the formation of double strand breaks in the *fbp1* region by facilitating access of the topoisomerase Rec12 [178], which make it a hotspot of recombination in meiosis. A similar regulatory situation of opening of epigenetic activation mediated by the transcription of lncRNAs was observed at the *ade6-M26* locus, also a recombination hotspot [166].

A lncRNA, *prt* (from phosphate repressing transcript), is involved in phosphate homeostasis in *S. pombe* through control of the expression of the *pho1* gene, orthologous to *PHO5* of *S. cerevisiae*, which encodes a secreted acid phosphatase involved in phosphate uptake. This is accomplished by activation of the *pho1* gene under phosphate starvation, a regulation carried out by the Pho7 transcription factor [105,179]. The lncRNA *prt*, which is transcribed from an upstream promoter on the same strand as *pho1,* participates actively in this regulation by repressing *pho1* transcription when phosphate is at sufficient levels. *Prt* transcription leads to RNAi-dependent histone methylation across the *pho1* locus, resulting in transient heterochromatinization. As observed for the meiotic gene *mei2*, under conditions of phosphate excess, Mmi1 interacts with *prt* through its DSR (Selective Removal Determinant) sequences to trigger its exosomal degradation, which is coupled to the termination of *prt* transcription [167]. Thus, disruption of *prt* DSR sequences results in *pho1* over-repression. Interestingly, mutations of the CTD domain of PolII do not affect the activity of the *prt* promoter, but affect the regulatory effect on *pho1* expression, indicating an involvement of the CTD in the control of *prt* [180]. The regulation of *Pho1* by *prt* requires its complete transcription. In mutants of the *erh1* gene, which encodes a small nuclear protein that interacts with Mmi1, *pho1* expression is derepressed [181]. This is because in this mutant, *prt* transcription is prematurely terminated, assigning Erh1 an antagonistic role in *prt* transcription termination, while Mmi1 promotes it. These data provide insight into the regulatory complexity of *prt*, in which other proteins involved in the progress and termination of its transcription participate.

Two other *pho1* adjacent genes, which also respond to the presence of phosphate, are also regulated by lncRNAs and form a regulon with *pho1*: the phosphate transporter gene *pho84* and the glycerophosphate transporter gene *tgp1* [105]. Mutations in the promoter of lncRNA *prt2* cause derepression of *pho84*, indicating the participation of *prt2* in the expression of this gene [168]. Interestingly, this regulatory alteration also affects expression of the adjacent downstream *prt1* and *pho1* genes, and therefore partially derepresses *pho1* as well, indicating a coordinated action of both lncRNAs in the same regulatory system. In addition, the gene *tgp1* is negatively regulated by its upstream lncRNA *nc-tgp1* [182]. As a mechanism of action, the transcription of *nc-tgp1* on the *tgp1* promoter increases the density of nucleosomes, blocking the access of activating transcription factor and silencing *tgp1*. However, rather than involving transient heterochromatin formation triggered by targeted lncRNA degradation, the regulation of *tgp1* by *nc-tgp1* RNA seems to be mediated by transcriptional interference. Reduced transcription of *nc-tgp1* allows *tgp1* expression during phosphate starvation, and loss of *nc-tgp1* induces *tgp1* even under repressive conditions. The polyadenylation site of *nc-tgp1* coincides with the binding element of the Pho7 regulator, so its transcription can negatively affect *tgp1* activation [169]. This lncRNA can be polyadenylated at an upstream site, resulting in a shorter transcript, and removal of this polyadenylation site also alters *tgp* expression. The choice of polyadenylation site is governed by the CTD of PolII, adding an additional novel control in the mechanism of action of *prt2*.

A combination of transcriptome analysis by RNA-seq and proteome analysis by mass spectrometry in response to stress allowed to associate changes in non-coding transcripts to specific protein changes [170]. To confirm this connection in one of them, deletion of the locus for the putative lncRNA *SPNCRNA.1164* resulted in reduced Atf1 protein levels and altered sensitivity to oxidative stress, indicating that *SPNCRNA.1164* is a lncRNA involved in the regulation of stress responses in this yeast. Interestingly, *SPNCRNA.1164* has two small antisense transcripts within the locus: *SPNCRNA.1165* and *SPNCRNA.1166/prl6*, which are longer than 200 nt and could participate in or even control its own function.

The elimination of Exo2/Xrn1 activity allowed the identification of numerous antisense lncRNAs whose increase in the mutant leads to the inactivation of their associated genes. This is the case of the catalase gene *ctt1* and its antisense lncRNA *XUT0794* [66]. The *ctt1* gene strongly increases its expression in the presence of H_2_O_2_, but the increase is significantly lower in the Exo2/Xrn1-deficient mutant, which shows higher levels of *XUT0794*. Expression of *XUT0794* is also stimulated by H_2_O_2_, suggesting a fine modulation of *ctt1* expression by its antisense RNA. The attenuation of *ctt1* by *XUT0794* is accompanied by low RNAPII-ser5 phosphorylation and involves the recruitment of histone acetylation HDAC machinery. This requires Set2-dependent H3K36me3 marks, as indicated by the similar effect produced by the absence of both HDAC and Set2 function.

#### 4.2.2. Sexual Cycle and Development

An interesting role recently identified for lncRNAs is the organization of nuclear structures [183]. The regulation of spatial positioning within the nucleus of eukaryotic cells is necessary for its proper functioning. The interaction between their components is facilitated by a correct compartmentalization in subnuclear domains or nuclear bodies [184]. This is the case of the lncRNA *meiRNA* in *S. pombe*, involved in the formation of a nuclear dot structure at the *sme2* locus, with its protein-binding partner Mei2 [185,186]. The *sme2* gene encodes two lncRNAs, *meiRNA-S* and *meiRNA-L*, that are required for Mei2 dot formation and play an important role in recognition of homologous chromosomes in meiosis [110,111]. The Mei2 structure promotes meiosis progression by sequestering free Mmi1, a factor involved in inducing selective degradation of meiosis-specific transcripts, thereby inhibiting their function [187]. Although the molecular details are not fully understood, it is assumed that the meiRNA functions as a lure to attract Mmi1. In fact, the localization of *meiRNA* at *sme2* depends on its association with Mmi1. Moreover, one of the multiple foci of Mmi1 in mitotic cells localizes to the *sme2* locus, and *meiRNA* overexpression promotes Mmi1 accumulation at the *sme2* locus even in the absence of Mei2 and reduces Mmi1 activity.

In a search of potential lncRNAs, those that passed several computational filters and were conserved in closely related species were identified, suggesting that they may have relevant functions [171]. The effects of deleting 12 intergenic lncRNAs on growth and development under different culture and stress conditions were analyzed. The mutant phenotype of one of them, *nc1995*, was noteworthy, as it initiates sexual development under conditions in which it should not, although it does not enter meiosis because it is not diploid. This phenotype is due to a repressing effect of *nc1995* on the expression of the *ste11* gene in the presence of nitrogen which causes deregulation of *ste11* in the mutant. The transition of *S. pombe* vegetative cells to meiosis and haploid ascospore formation is triggered by nutrient starvation. This process, which involves conjugation between h+ and h- cells and subsequent entry into meiosis, is regulated by the product of the *ste11* gene [188].

Expression of *ste11* is also negatively regulated by the lncRNA *rse1*, which is divergently transcribed from the *ste11* promoter [172]. Its mechanism of action is in *cis*, functioning as a scaffold to recruit a repression complex that promotes SET3C-dependent histone deacetylation. Upstream to *rse1* there is a smaller ncRNA, *rce1*, probably also a lncRNA, that could interfere with *rse1* transcription. Another lncRNA that acts in the control of the transition from mitosis to meiosis is *mamRNA*, which acts as a scaffold in a specific nuclear body to facilitate the interaction between the proteins Mmi1 and Mei2 and their mutual inhibition [173]. In this interaction, Mmi1 downregulates Mei2, inducing its ubiquitinylation by another complex, while Mei2 removes part of Mmi1, with the remainder being available to facilitate the degradation of the lncRNA *meiRNA*—already mentioned above—and meiosis mRNAs. Under conditions of meiosis stimulation, there is a greater amount of *meiRNA*, which sequesters Mei2 and Mmi1, so that it stimulates the translation of the mRNAs of the genes necessary to enter meiosis.

The detection of RNAs by the Mmi1 protein determines their degradation by nuclear exosomes. In the search for Mmi1 RNA targets, lncRNA *nam1* (from non-coding RNA associated with Mmi1) was identified, which also participates in the regulation of sexual differentiation [174]. The binding of Mmi1 to *nam1* not only promotes its degradation but also stops its transcription when it binds to the nascent RNA, thus activating the downstream gene *byr2*, in whose expression the complete transcription of *nam1* interferes negatively. Byr2 is a mitogen-activated MAPKKKK that plays a key role in sexual differentiation. In addition, Mmi1 arrests the transcription of other lncRNA genes in pericentromeric regions, contributing to their silencing as heterochromatin. In the mechanism of action of the inactivation of *byr2* by the upstream transcription of *nam1*, an important role is played by a cryptic intron found in the sequence of *nam1* overlapping with *byr2*, which forms a scaffold for recruiting splicing machinery and other proteins, such as Pir2, which plays various roles in RNA metabolism [189]. As a result, chromatin-modifying activities involved in gene silencing are formed, leading to gene repression. The same mechanism seems to operate for other genes in *S. pombe*, such as the regulation of *Pho1* by the lncRNA *prt*, mentioned above.

In *S. pombe*, about 70 genes affecting its chronological lifespan have been identified, some of them related to ribosome function [190]. One lncRNA, *aal1* (from aging-associated lncRNA), is expressed in quiescent cells and lengthens their lifespan by attenuating their translation activity [175]. This action is exerted in *trans*, since its ectopic overexpression further extends the lifespan, reducing the number of ribosomes, while its deletion shortens it by increasing its translational activity. Its mechanism of action consists of binding to the mRNA *rirpl1901*, which encodes a ribosomal protein, reducing its availability for ribosome formation. In congruence, *aal1* is mainly detected in the cytoplasm, with a tendency to be associated with ribosomes. Therefore, this lncRNA plays an important evolutionary role, as it reduces the translational activity of cells when they are at rest, waiting for suitable conditions to resume their life cycle.

### 4.3. Other Yeasts

Research on specific lncRNAs is rare in other yeasts. In the study conducted in *P. pastoris*, mentioned in Section 2.1, three lncRNAs that stood out for their changes in expression due to overexpression of phospholipase A2 and the presence of methanol, *TCONS_00004115*, *TCONS_00000958*, and *TCONS_00003668*, were deleted for analysis of their possible function [50]. When analyzing the effects on possible target genes, in *cis* based on physical proximity or in *trans* based on coexpression analysis under the tested conditions, only changes in some of the possible target genes were found in the *TCONS_00004115* mutant, related to small GAPase signal transduction, presumably involved in the adaptation to the physiological conditions tested.

### 4.4. Dimorphic Fungi

LncRNAs play also important roles in the regulatory mechanisms of dimorphic fungi. Many of them are pathogenic in animals, and lncRNAs can modulate key processes in their pathogenic activity, such as dimorphic transition, virulence, stress response and host adaptation. Their study may open new perspectives for understanding the phenotypic plasticity of these fungi and could reveal new therapeutic targets against fungal infections. Table 4 lists the lncRNAs that have received most attention in this group of fungi and whose mechanisms of action are summarized in this section.

#### 4.4.1. *Ustilago maydis*

Some of the noncoding RNAs identified in the *U. maydis* cDNA libraries show differential expression at different stages of development and may be associated with the pathogenesis process. This prediction was confirmed for one of them, called *ncRNA1* [70]. Deletion of the gene for this putative lncRNA does not produce detectable phenotypic changes, except for a reduction in virulence on maize. The mechanism by which *ncRNA1* is involved in pathogenesis remains to be elucidated, but its deletion results in increased expression of the *um12316* gene, which encodes a secreted protein [194]. Moreover, *ncRNA1* partially overlaps in antisense orientation with gene *um02150*, which itself possesses an antisense RNA, *as-um12316*, and whose function could also be associated with pathogenesis [195]. Engineered-induced *as-um12316* levels in haploid cells, in which it is normally barely expressed, results in double levels of its complementary *um12316* mRNA, presumably by stabilization in two-stranded forms, which could be experimentally detected. However, doubling of *um12316* content does not lead to increased amounts of the encoded protein, probably because the additional mRNA is not available for translation.

Another antisense RNA investigated in detail in *U. maydis* is *as-ssm1* [108]. The complementary gene, *ssm1*, encodes a mitochondrial seryl-tRNA synthetase, that catalyzes the transfer of L-serine to tRNA and its deletion causes cell lysis, making it a necessary gene for the integrity and viability of the fungus. The *ssm1* gene is induced in the dikaryotic stage, and its ectopic expression in haploid cells slows growth, reduces virulence and alters mitochondrial activity, as evidenced by reduced oxygen consumption. Similarly to what was observed with the *um12316* gene, *ssm1* mRNA levels increase and two-stranded *as-ssm1/ssm1* forms are detected, without changing the amount of Ssm1 protein. The data suggest a regulatory role of this antisense RNA reducing mitochondrial function in the teliospores, which may be involved in its dormancy state. Thirty-three other antisense mitochondrial gene transcripts, with an average length of 720 nt, have been detected, making it likely that *as-ssm1* functions in coordination with other antisense transcripts to reduce mitochondrial activity in a more general mechanism associated with the dormancy state in this group of fungi.

Telomerase RNA production varies in the different eukaryotic kingdoms, with differences in processing or in the transcription machinery involved, polymerases II in animals or some fungi (see Section 4.1.4) or polymerase III in plants. In *U. maydis* the telomeric RNA *TER* is not derived from a separate transcript, but from a long UTR region of the *emi1* gene transcript, which encodes the “early meiotic induction protein” [191]. The *emi1* mRNA is subject to typical capping, polyadenylation and intron splicing, and may be subject to alternative maturation, including processing to produce the 1291 nt *TER*.

#### 4.4.2. *Candida auris*

Dimorphism in *Candida* plays an important role in its pathogenicity. Thus, the filamentous form is more effective as an invasive agent than the yeast form during infection, although the unicellular form is more effective for dispersal. Therefore, in addition to other factors, as biofilm formation or enzymes or toxins secretion, the control of the yeast/hyphae switch plays an important role in its biological cycle [196]. In a screening of C. *auris* mutants obtained by insertion with a transposon, a mutant was identified that develops only in the filamentous form [192]. The mutant is affected in a lncRNA, called *DINOR* (from DNA damage-inducible non-coding RNA), and has higher levels of DNA damage due to the DNA checkpoint kinase RAD53 being hyperphosphorylated. This phenotype is related to the filamentous growth-inducing effect of DNA alkylating agents, which induce *DINOR* expression. This lncRNA modulates the response to different stress agents, including the presence of certain antifungals, H_2_O_2_, SDS, or macrophages. These stressing conditions upregulate *DINOR*, indicating that this lncRNA integrates different stress signals, as well as having a role in the maintenance of genome integrity.

#### 4.4.3. *Cryptococcus neoformans*

*C. neoformans* is another pathogenic yeast in which the morphological change from yeast to hyphae plays a relevant role in pathogenesis, in addition to other processes, such as the sexual cycle. A major role in this morphological transition is played by the transcription factor Znf2 [197]. An *Agrobacterium*-mediated insertional mutation screening in this species allowed the identification of a mutant with a yeast-like phenotype, similar to that shown by the mutant lacking Znf2. The Ti insertion in the mutant affects a gene for a lncRNA located 2.5 kb upstream of *ZNF2*, which was named *RZE1* [193]. This lncRNA is functionally restricted to its own nucleus, as revealed in a heterokaryon assay, and exerts its function mainly in *cis*. Transcriptomic and RNA localization analysis showed that the mutant lacking *RZE1* has lower levels of *znf2* mRNA and it accumulates in greater proportion in the nucleus, so that this lncRNA facilitates both its transcription and its passage to the cytoplasm, allowing a sufficient amount of functional Znf2 protein. *RZE1* transcript is found in all subspecies of the *Cryptococcus neoformans* species complex, indicating a conserved role in this pathogenic fungal group.

#### 4.4.4. *Ustilaginoidea virens*

In the study of lncRNAs by RNA-seq carried out in *U. virens* (see Section 2.3), one of the identified lncRNAs, Uvlnc001515, was studied in more detail [53]. This was renamed *UvlncNAT-MFS* because its 3′ end is antisense to the 3′ end of transcript of gene *UvMFS* (*Uv-07373*), which encodes a putative transporter of the Major Facility Superfamily, probably involved in zinc transport. The two transcripts showed similar expression patterns throughout different stages of rice infection, and parallel transcript levels were also found in strains silenced or overexpressing *UvlncNAT-MFS* or *UcMFS*, evidencing an interdependent expression. Either repression or overexpression of both transcripts produced abnormal fungal growth, indicating the importance of fine-tuned regulation of their transcripts. However, none of these changes led to alterations in pathogenesis. The detail of the mechanism of action of the regulation of *UvlncNAT-MFS* on *UcMFS* is unknown, except that it was demonstrated that they form a duplex RNA structure in vivo and they co-localize on in situ hybridizations.

### 4.5. Filamentous Fungi

Although the morphological complexity and metabolic versatility of filamentous fungi make them models of great interest for research, and some of them are very well-stablished research objects, studies on specific lncRNAs in filamentous species are not abundant, although their number continues to grow. Major findings on specific lncRNAs in these fungi, listed in Table 5, are summarized below.

#### 4.5.1. *Neurospora crassa*

One of the aspects in which *Neurospora crassa* has stood out as a research model is the study of circadian rhythms [212]. Several lncRNAs have been identified that modulate the expression of genes involved in the regulation of the oscillation of the circadian machinery. One of the best known is the antisense transcription of the *frq* (*frequency*) gene, a central component of the circadian clock. A lncRNA antisense to the *frq* gene, termed *qrf*, regulates *frq* sense mRNA expression through transcriptional interference [199]. Subsequent work that has deepened on the mechanism of action observed that *qrf* transcription initially promotes *frq* expression by facilitating a suitable chromatin environment but subsequently leads to its inactivation by heterochromatinization [200]. The transcription of *qrf* contributes to modulating the amplitude and duration of *frq* expression, whereas *frq* transcription inhibits *qrf* expression and drives the antiphase rhythm of both transcripts. Thus, mutual inhibition of *frq* and *qrf* transcription forms a double negative feedback loop that is part of the core of circadian rhythm functioning [198]. In turn, *qrf* is not essential for circadian rhythmicity, and its transcriptional rhythm is mediated by the morning-specific repressor CSP-1 [213]. This scenario provides an example of how lncRNAs can act as regulators of biological oscillators through interference mechanisms.

#### 4.5.2. *Trichoderma reesei*

In different strains of *T. reesei*, known for their capacity to produce cellulases, a lncRNA called *HAX1* has been found whose length varies in different strains, being longer in hypercellulolytic strains [201]. A correlation was observed between the expression of this lncRNA and cellulolytic activity, the latter being increased in strains in which *HAX1* is overexpressed, which confers biotechnological interest to this lncRNA. The mechanism of action is by interacting with a set of specific regulators of these enzymes, as seems to be the case for the xylanase activator Xyr1 [112]. It was observed that Xyr1 can inhibit its own transcription by a feed-back mechanism; a regulatory model has been proposed in which *HAX1* physically interacts with Xyr1, reducing this retroinhibition and increasing its availability to increase the expression of its target genes, including the cellulase genes *cbh1* and *cbh2*.

#### 4.5.3. *Fusarium fujikuroi* and *Fusarium oxysporum*

**Figure 3:**
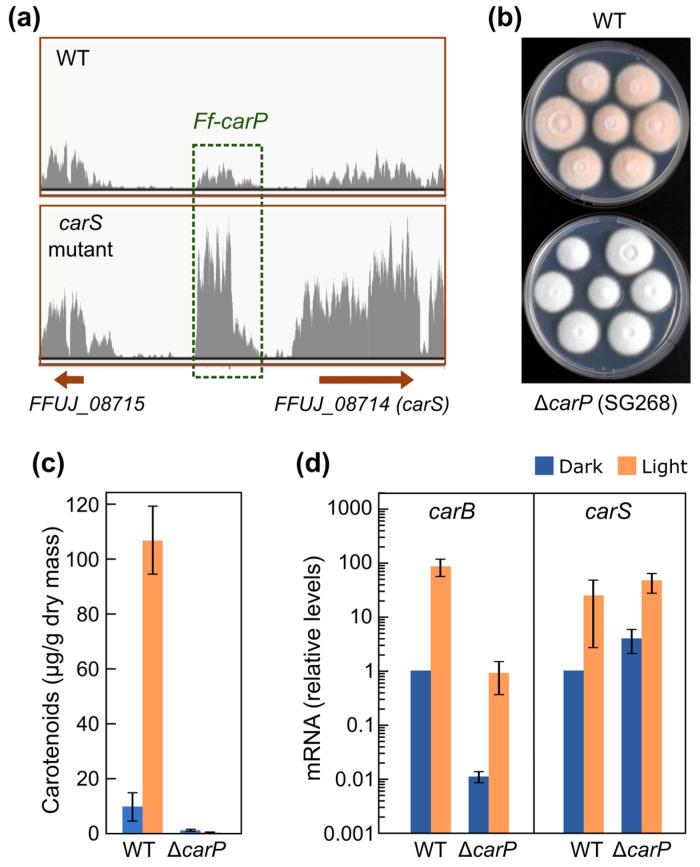
Identification and effect of deletion lncRNA *carP* in *Fusarium fujikuroi*. (**a**) Transcript readings according to RNA-Seq data in the genomic region between the *Ffuj_08715* and *carS* (*Ffuj_08714*) genes in the wild-type (WT) and *carS* mutant SG39 of *F. fujikuroi*. The readings were represented with the IGV program. The *carP* transcript is marked with a dashed box. (**b**) Colonies of the wild type and a representative Δ*carP* mutant grown on minimal medium for 1 week under light. (**c**) Carotenoid content in the wild-type and the same Δ*carP* mutant grown for 1 week in the dark (blue bars) or under light (orange bars). (**d**) Transcript levels for the carotenoid gene *carB*, and neighbor gene *carS* in the same strains grown in the dark or exposed for 1 h to light. RT-qPCR data show the mean and standard error of three independent experiments. Relative mRNA levels refer to the mRNA content of the wild-type strain in darkness. Adapted from Parra-Rivero et al. [202].

Carotenoid synthesis in *Fusarium fujikuroi* and *Fusarium oxysporum* is activated by light and certain stress conditions, and is negatively regulated by a protein of the RING finger family, called CarS [214]. Mutants with a loss of function of the CarS protein show strong pigmentation due to upregulated expression of the carotenoid pathway genes. Transcriptomic analyses of the effect of light and the *carS* mutation, which affects hundreds of genes [215], detected upstream of the *carS* gene a lncRNA in both species, called *Ff-carP* and *Fo-carP* [202]. Deletion of either of both lncRNAs resulted in a sharp decrease in the mRNA levels of carotenoid biosynthetic genes and an albino phenotype (Figure 3). In addition to lacking assignable coding function, the non-coding nature was evidenced by the absence of significant overlapping ORFs when comparing *Ff-carP* and *Fo-carP*, due to the presence of small deletions/insertions, despite high sequence conservation and coincidence in the phenotypic effects of their mutations.

*Ff-carP* and *Fo-carP* are transcribed from the same strand than *carS* in their respective genomes, and the deletion of any of them upregulates *carS* mRNA levels, so it is proposed that the action of these lncRNAs is at the level of interfering with *carS* transcription. Consistently, overexpression of *carS* in *F. fujikuroi* results in an albino phenotype similar to that produced by *Ff-carP* deletion [216]. In support, when *Ff-carP* gene was reintroduced into the deletion mutants the carotenoid synthesis phenotype was only recovered when inserted upstream of *carS* [203]. In this work, the effect of its deletion on the transcriptome was also investigated and found to affect the transcript levels of several hundred genes. Most changes could be explained by its action on *carS*, but strong alterations were found in the expression of some genes that were not affected by the *carS* mutation. So, it is not excluded that *Ff-carP* may play other regulatory functions in *trans* in addition to its regulatory effect on *carS* in *cis*.

#### 4.5.4. *Fusarium graminearum*

Several lncRNAs related to development and pathogenesis have been investigated in *Fusarium graminearum* (*Gibberella zeae*), a pathogen of maize, also infecting wheat and barley. In this fungus, the antisense lncRNA *GzmetE-AS*, which is transcribed from the complementary strand of the *GzmetE* gene, encoding a homoserine O-acetyltransferase involved in sexual development and pathogenesis, has been described [204]. The expression of *GzmetE*, low during conidiation and high at the end of the sexual cycle, is opposite to that of *GzmetE-AS*. In confirmation, an inverse correlation between both transcripts was observed in strains overexpressing this lncRNA or with premature termination in its 5′ region. In fact, strains lacking *GzmetE-AS* by the presence of the 5′ terminator show a phenotype similar to that of *GzmetE*-overexpressing strains, including an increased production of sexual structures. The regulation of this antisense lncRNA is carried out through the destruction of *GzmetE* mRNA by the RNA interference (RNAi) system, as evidenced by the fact that the reduction in *GzmetE* mRNA in *GzmetE*-AS-overexpressing strains is not observed in mutants lacking RNAi Dicer proteins.

The synthesis of deoxynivalenol (DON), a mycotoxin contaminating food and cereals infected by this fungus, is regulated by different proteins, including transcription factors such as Tri6 and Tri10. The study of their function by RNA-seq analysis of *tri6* and *tri10* mutants allowed the identification of two non-coding RNAs involved in the regulation of *tri5*, which encodes a key enzyme in the synthesis of these metabolites [205]. On the one hand, a *tri5* antisense RNA is transcribed, which negatively regulates *tri5* levels, as evidenced by its reduction when this antisense RNA is overexpressed. On the other hand, *tri5* is regulated by the lncRNA *RNA5P*, which is transcribed in the same direction in its promoter region. Loss of *RNA5P* transcription results in increased expression of *tri5*, pointing to *RNA5P* as a negative regulator interfering with *tri5* transcription. Consistent with this hypothesis, *RNA5P* exerts its action in *cis*, since its ectopic overexpression has no consequences on *tri5* expression.

In a previous study, another lncRNA involved in the regulation of DON synthesis was found through its effect on the *Fgsp1* gene, which encodes a protein of the sugar transporter family. Mutation of this gene has pleiotropic effects, including alterations in the sexual cycle (in ascospore release), virulence, and DON synthesis. Interestingly, a similar phenotype is shown by mutants lacking a lncRNA called *lncRsp1*, detected in the promoter region of *rsp1* and transcribed from the same strand [206]. Both *Fgsp1* and *lncRsp1* negatively regulate the expression of several DON biosynthesis genes, *TRI4*, *TRI5*, *TRI6*, and *TRI13*. Loss of lncRsp1 transcription reduces *rsp1* mRNA levels, so by an unknown mechanism its expression favors transcription from the same promoter.

A study of alternative splicing and polyadenylation in *F. graminearum* led to identify the lncRNA *LncRsn*, located between coding genes *FgSna* and *FgPta* [207]. *FgSna* contains a Longin domain, usually associated with vesicle trafficking, and FgPta is a phospholipid-transporting ATP-ase. *LncRsn* is antisense to *FgSna* and is transcribed from the same strand as *FgPta*, and its deletion reduced the number of perithecia and the number of ascospores [208]. However, conidia formation and germination were not affected by the deletion, supporting that is not necessary for asexual reproduction. *LncRsn* has an important role in the infection of wheat because the deletion mutant showed a lower disease index and reduced length of the lesions in the coleoptiles and in the corn silks. This lncRNA is involved in the regulation of the biosynthesis of DON, as the mutants produced double the amount of DON than the wild type. When the expression of the flanking genes were analyzed by RT-qPCR, *FgSna* and *FgPta* were found to be strongly and partially downregulated in the mutant, respectively, reinforcing the regulatory function of this lncRNA.

#### 4.5.5. *Cochliobolus heterostrophus*

Dark pigmentation in the maize pathogen *Cochliobolus heterostrophus*, due to melanin accumulation, increases under illumination or hyperosmotic stress, and is controlled by the transcription factor Cmr1, whose gene expression is regulated by the mitogen-activated protein kinases (MAPKs) Chk1 and Mps1 [209]. Interestingly, the *cmr1* gene is transcribed from both strands, giving rise to sense and antisense *cmr1* transcripts, both of which are upregulated by Chk1 and Mps1. An epigenetic regulation at the level of the chromatin organization of *cmr1* expression was proposed, but the involvement of the *cmr1* antisense transcript in this mechanism, or its possible neutralizing role on the sense *cmr1* mRNA, has not been elucidated.

#### 4.5.6. *Metharhizium robertsii*

Strand-specific ARN-seq analysis in the insect pathogen *Metarhizium robertsii* led to the identification of 1655 lncRNAs in germinated conidia after a heat treatment at 40 °C, of which 1081 were differentially expressed [87]. Expression level in the conidia of one of them, *mrLncRNA1,* increased ca. 200-fold after the heat treatment, and was chosen for a more detailed study. Deletion of this lncRNA resulted in a reduction in germination of 31% compared to the wild-type and complemented strains, suggesting that *mrLncRNA1* is involved in conidial thermotolerance through an unknown mechanism.

#### 4.5.7. *Phytophthora infestans*

*Phytophthora infestans*, the causal agent of potato late blight disease, produces important economic losses [217]. For this reason, much effort has been devoted to investigating the mechanism of pathogenesis by this fungus. In one of the works, the analysis of a *P. infestans* cDNA library enriched with sequences regulated during potato infection led to the identification of a new gene family of predicted non-coding sequences named *Pinci1* (from *P. infestans* non-coding infection-specific family 1). This family consists of more than 400 *Pinci1*-like copies, with high similarity to each other, organized in clusters at more than 90 locations in the *P. infestans* genome [210]. The coding features were investigated in detail in two of the *Pinci1* sequences, *Pinci1-1* and *Pinci1-5*, about 700 nt in length. Lack of coding function was checked by bioinformatic means (Testcode algorithm) and confirmed by the lack of detection of FLAG-tagged proteins after expression of three putative FLAG-tagged *Pinci1*-ORFs. They are thus a family of related non-coding polyadenylated transcripts that are upregulated during potato infection. However, their possible role in the mechanism of pathogenesis remains unknown.

#### 4.5.8. *Pyricularia oryzae*

RNA-seq data in hyphae and conidia in *P. oryzae* showed differential expression for the *MGG_15773* gene, with much higher mRNA levels in conidia than in hyphae. The lncRNA *G003973*, which is transcribed from the same strand upstream of *MGG_15773*, in its promoter region, shows an opposite pattern of expression, consistent with this lncRNA being a negative regulator of its transcription [59]. Deletion of *G003973* resulted in increased expression of the *MGG_15773* gene in three independent mutants, which showed slower development of their colonies than the control strain, indicating that lncRNA *G003973* plays a regulatory role on hyphal development through attenuation of transcription of its neighboring gene *MGG_15773*.

#### 4.5.9. *Rhizopus delemar*

In *Rhizopus delemar*, an opportunistic pathogen that causes mucormycosis, weighted gene co-expression network analysis (WGCNA) was done to identify lncRNAs involved in the germination of conidia [211]. Most of the cell wall modifying enzymes were predicted to interact with lncRNAs. For example, lncRNAs *MSTRG.10396.1* and *MSTRG.13812.1* were foreseen to interact with *RO3G_03340* mRNA coding for a chitin β-1–6-glucanosyl transferase, and lncRNAs *MSTRG.13812.1* and *MSTRG.13812.1* were predicted to interact with *RO3G_01689* and *RO3G_04985* mRNAs, coding for α-mannosyltransferases. Although such interactions remain to be experimentally demonstrated, the one with the highest probability (lowest free energy) is included in Table 5.

#### 4.5.10. *Verticillium dahliae*

In the analysis on the occurrence of lncRNAs involved in pathogenesis in *Verticillium dahliae* (see Section 2.4), identified by changes in their expression patterns in mutants of genes for cell wall lytic enzymes, the involvement of two intergenic lncRNAs was investigated through the effect of their overexpression [58]. Strains overexpressing *LNC_01522* (Gene id. *XLOC_006536*) showed a 10-fold increase in the expression of the *VDAG_09366* and *VDAG_01782* genes, encoding respectively for an endo-polygalacturonase and a pectin esterase, and a 4-fold increase in the *VDAG_01205* gene, encoding an endoglucanase. These strains produce more severe leaf damage, demonstrating the participation of *LNC_01522* in cotton plant pathogenesis. On the other hand, overexpression of *LNC_000201* (Gene id. *XLOC_000836*) produced a 6-fold increase in the expression of the *VDAG_09366* gene, encoding for an endo-polygalacturonase, but this alteration was not sufficient to show an apparent change in virulence.

In a parallel work, other lncRNAs were identified showing expression changes during the infection process and overexpressed to test the consequences on pathogenesis [82]. Virulence assays with the engineered strains showed that overexpression of *lncRNA012077* resulted in more severe disease symptoms compared to the wild type strain, whereas overexpression of *lncRNA009491* or *lncRNA007722* resulted in reduced virulence, the effect being more pronounced in the case of *lncRNA009491*. However, no apparent effects were found in the case of other lncRNAs. These results indicate a collective action between numerous lncRNAs in the infection process, some acting as positive and others as negative regulators, with a more relevant participation for some of them.

## 5. Conclusions and Future Prospects

Global transcriptomic analysis techniques have enabled new approaches to the study of lncRNAs by contrasting transcriptomes with annotated genomes. Over the last decade, the number of lncRNAs investigated in fungi has exploded, most of which have regulatory functions. From their location respective to protein coding genes, they are basically distributed into two types, intergenic and antisense lncRNAs. The cases investigated in more detail reveal a wide diversity of mechanisms of action, greatly increasing the complexity of gene regulatory systems in fungi. Interference with transcription or promotion of changes in chromatin structure are frequent in intergenic lncRNAs, and interference with the mRNA with which they overlap is a predictable consequence in antisense lncRNAs, but other studies reveal numerous variations on these themes, including facilitation of recruitment of other effector proteins or *trans*-acting effects.

The development of new techniques and the improvement of CRISPR-directed mutagenesis methodologies is allowing more ambitious strategies in the study of lncRNA functions. An example is the massive study of the function of 141 intergenic lncRNAs carried out in *S. pombe* by systematic deletion, allowing phenotypes to be found either visually or by microscopy or flow cytometry for almost 60% of the lncRNAs analyzed [218]. This work also showed the diversity of biological processes to which they are associated, demonstrating that fungi use this regulatory tool in a massive and generalized way, considerably increasing the sophistication of their gene regulation systems. In addition, the efficiency of regulation by lncRNAs opens new perspectives for their biotechnological applications. In *S. pombe*, a *pho1* reporter gene regulated by the lncRNA nc-*tgp1*, whose transcription is regulated by the thiamine-activatable *nmt1* promoter, has been tested [219]. This hybrid regulatory system allows for strong and rapid induction of any gene of interest, enabling the adjustment of its transcription through the concentration of thiamine in the medium. Another case, already mentioned in Section 4.1.1, is the increase in the yield of heterologous protein production by *S. cerevisiae* due to the deletion of certain intergenic lncRNAs [123]. These are just two examples of the vast field of possibilities opened by the study of the mechanisms of action of lncRNAs, with implications in such diverse areas as biotechnological production or biocontrol and treatment of fungal diseases.

Despite advances in their identification, the study of lncRNAs in fungi is an area of research with significant constraints. First, knowledge about the precise function of many of the lncRNAs that have been investigated remains limited. This is largely due to their diversity and the difficulty in identifying their molecular targets and mechanisms of action, especially in the case of intergenic lncRNAs. Their study faces several challenges, including their low evolutionary conservation, as they tend to be very species-specific, making extrapolation of results difficult, and their often low or transient expression (partially resolved by the use of mutants of the degradation machinery), making it difficult to distinguish functional lncRNAs from transcriptional noise. Progress in this field is expected to be enhanced using new technologies, e.g., in structural biology, to visualize how lncRNAs fold and interact with target proteins, or in bioinformatics, with the growing support of artificial intelligence, to predict the formation of secondary structures or interaction with other molecules with increasing reliability. Methodologies for identifying lncRNA partners in which advances are expected include high-resolution in vivo RNA imaging, identification of RNA-binding proteins (ChIRP), capture hybridization analysis of RNA targets (CHART), or different purification or immunoprecipitation techniques), fostered by the increasing importance of lncRNAs in biomedicine [220]. Advances are also expected in single-cell omics techniques, which should allow the analysis of expression variability in heterogeneous cell populations, of special interest in yeasts.

An important aspect in lncRNA research is the assumed absence of coding functions. Demonstrating this absence requires the use of unusual techniques, such as ribosome profiling, which, due to their technical complexity, are not often addressed in lncRNA studies. However, just as there is pervasive transcription, there is also pervasive translation. This is not unexpected, since XUTs are subject to NMD, implying that they are recognized by ribosomes [221]. Pervasive translation has been confirmed in recent studies in *S. cerevisiae*, which show that many lncRNAs can be recognized by ribosomes and give rise to small proteins [222,223], expanding the proteome of this yeast and blurring the boundary between coding and non-coding RNAs. The study of the possible functions of these proteins is an experimental challenge which is beginning to be addressed. An important aspect of these novel peptides is that they may co-evolve with other proteins in the genome, and they may play an evolutionary role in the generation of new coding genes [224,225,226].

The available data on lncRNAs in fungi is heavily biased towards yeasts, mostly due to their experimental advantages, especially outstanding in *S. cerevisiae.* It is expected that in the future there will be increasing attention to the study of lncRNAs in filamentous fungi for different reasons. Many of their species are particularly relevant as pathogens, especially in agriculture, or have biotechnological interest due to their metabolic versatility or their use as biocontrol agents. They also stand out for their ability to adapt to changing environments and respond to adverse conditions. In addition, they have more complex morphologies and development programs than yeasts, not only due to the formation and branching of hyphae, but also to the processes of sporulation or fruiting body formation [227]. All of this is reflected in the larger size of their genomes, often in the range of 40–50 Mb, while yeast genomes are typically around 10–15 Mb in size. Therefore, greater regulatory complexity mediated by lncRNAs can be expected in filamentous fungi, making research in this area particularly promising.

## Figures and Tables

**Table 1:** Examples of global identifications of intergenic and antisense lncRNAs in wild-type fungi.

Species	IG lncRNAs ^a^	AS lncRNAs ^b^	Technology ^c^	Refs.
*Saccharomyces cerevisiae*	77	21	T	[38]
	234	193	T	[23]
	667	367	C	[25]
	487	ND	S	[26]
	ND	1103	S	[27]
*Schizosaccharomyces pombe*	427	37	S, T	[48]
	338	546	S	[49]
*Pichia pastoris*	36	168	S	[50]
*Ustilago maydis*	ND	210	C	[51]
	2414	2624	S	[52]
*Ustilago hordeum*	1206	1606	S	[52]
*Sporisorium reilianum*	1776	1949	S	[52]
*Ustilaginoidea virens*	1084	566	S	[53]
*Neurospora crassa*	462	477	S	[54]
*Aspergillus flavus*	939	1124	S	[55]
*Magnaporthe oryzae*	1286	980	S	[56]
*Botrytis cinerea*	945	743	S	[57]
*Verticillum dahliae*	1432	1533	S	[58]
*Pyricularia oryzae*	2478	508	S	[59]
*Fusarium graminearum*	1054	1040	S	[60]

^a^ IG: intergenic (not overlapping with a coding sequence). ^b^ AS, antisense. ND: not determined. ^c^ C, cDNA sequencing. T, Tile arrays. S, RNA-seq.

**Table 2:** Specific lncRNAs investigated in *S. cerevisiae*.

lncRNA	Target	Location ^a^	Cellular Function	Mechanism of Action ^b^	Refs.
*SRG1*	*SER3*	I, US	Serine biosynthesis	C, chromatin remodeling	[104,105,106]
*usURA2*	*URA2*	I, DS	Pyrimidine biosynthesis	C, TSS selection	[116]
Attenuated transcripts	*IMD2*	I, DS	Inosine biosynthesis	C, TSS selection	[117]
*HRA1*	*DRS2?* *18S RNA?*	A of *DRS2*	Golgi protein transport? 18S RNA maturation?	T, ND	[38]
*GAL10 ncRNA*	*GAL1*, *GAL10*	A of *GAL10* O of *GAL1*	Galactose utilization	C, chromatin remodeling	[118,119]
*PHO5* antisense	*PHO5*	A	Phosphate utilization	C, chromatin remodeling	[102,103]
*PHO84* antisense	*PHO84*	A	Phosphate utilization	C, chromatin remodeling	[101,102]
*ncASP3*	*ASP3*	O	Nitrogen metabolism	C, chromatin remodeling	[120]
*TBRT*	*TAT1* *BAP2*	A I, UA	Amino acid transport	C, chromatin remodeling	[121]
*CUT60*	*ATP16*	I, US	Mitochondrial function	C, transcription promotion	[122]
*SUT067* *SUT433* *CUT782*	ND	ND	Energy metabolism Translation activity	ND	[123]
*SUT125*, *SUT126*, *SUT035*, *SUT532*	ND	ND	Diverse functions	T, unknown	[124]
*FAR1* antisense *CTF4* antisense *TAF2* antisense	*FAR1* *CTF4* *TAF2*	A	Cell cycle TFIID function (*TAF2*)	C, ND	[125]
*PET10* antisense *CLD1* antisense *MOH1* antisense *SSH3* antisense	*PET10* *CLD1* *MOH1* *SSH3*	A	Expressed in quiescent cells	C, transcription interference	[126]
*CDC28* antisense	*CDC28*	A	Cell cycle and stress	C, chromatin remodeling	[127]
*lnc9136*	Hsl1p and Gin4p?	ND	Cell cycle and ethanol stress. Translation	T, protein decoy?	[128]
*IRT1* *(RME1)*	*IME1*	I, US	Meiosis entry	C, transcription interference, chromatin remodeling	[129,130,131]
*IRT2*	*IRT1, IME1*	I, US	Meiosis entry	C, chromatin remodeling	[132]
*RME2*	*IME4*	A	Meiosis entry	C, transcription interference. Chromatin remodeling?	[100]
*RME3*	*ZIP2*	A	Meiotic recombination	C, chromatin remodeling	[133]
*ICR1* *PWR1*	*FLO11* *ICR1*	I, US A	Pseudohyphal growth	C, chromatin remodeling, transcription interference	[134]
*SUT719*	*SUR7*	A	Repressed by α-pheromone	C, ND	[135]
*EUC1*	*ECM3*	I, US	Cell wall	C, chromatin remodeling	[136,137]
*SUT228*	*PIR3*	I, US	Cell wall	ND	[137]
*SUT169*	*SPS100*	A	Cell wall	mRNA isoform regulation	[137,138]
*TIR1axut*	*TIR1*	A	Cell wall	C, chromatin remodeling	[44,137]
*pHO-lncRNA*	*HO*	I, US, O	Mating type switch	C, transcription interference	[139]
Ty1 antisense	Ty1 transposon	A	Transposon control	C, chromatin remodeling	[107]
*cenRNAs*	CENP-A, CENP-C and others	Centromeres	Kinetochore formation	T, protein recruiting	[140]
*TLC1*	Telomerase	NR	Telomerase function	T, protein recruiting	[141]
*TERRA*	Telomerase proteins	Subtelomeric	Telomerase function	T, telomerase inhibition	[109]
*SUT457*	Telomere ends?	NR	Telomere maintenance	T, ND	[142]

ND: Not determined. NR: Location not related to target. ^a^ A, antisense of a coding sequence. I, intergenic: US, upstream of target in the same sense; DS, downstream of target in the same sense; UA, upstream of target in opposite sense. O, overlapping in the same sense. ^b^ C: *cis*-acting lncRNA; T: *trans*-acting lncRNA.

**Table 3:** Specific lncRNAs investigated in *S. pombe*.

lncRNA	Target	Location ^a^	Cellular Function	Mechanism of Action ^b^	Refs.
*mlonRNA-a* *mlonRNA-b* *mlonRNA-c*	*fbp1*	I, US	Gluconeogenesis	C, chromatin remodeling	[165,166]
mlonRNAs	*ade6-M26*	I, US	Purine synthesis	C, chromatin remodeling	[166]
*prt*	*pho1*	I, US, O	Phosphate utilization	C, chromatin remodeling	[167]
*prt2*	*pho84* *tgp1*	I, US, O I, US	Phosphate utilization	C, transcription interference? C. ND	[168]
*nc-tgp1*	*tgp1*	I, US	Phosphate utilization	C, chromatin remodeling, transcription interference	[169]
*SPNCRNA.1164*	atf1	ND	Oxidative stress	ND	[170]
*XUT0794*	*ctt1*	A	Oxidative stress	C, chromatin remodeling	[66]
*nc1995*	*ste11*	NR	Sexual development	T, ND	[171]
*rse1* *rce1*	*ste11* *rse1*	I, UA I, US	Sexual development	C, chromatin remodeling C, transcription interference?	[172]
*meiRNA*	*sme2*, Mei2	I	Nuclear organization	C (at own *sme2* locus) T, protein recruiting	[110,111]
*mamRNA*	Mmi1, Mei2	NR	Nuclear organization	T, protein recruiting	[173]
*nam1*	*byr2*	I, US	Sexual development	C, chromatin remodeling	[174]
*aal1*	*rirpl1901*	NR	Chronological lifespan	T, binding target mRNA	[175]
*TER1*	Telomerase	NR	Telomerase function	T, protein recruiting	[161,162]

ND: Not determined. ^a^ A, antisense of a coding sequence. I, intergenic: US, upstream of target in the same sense; UA, upstream of target in opposite sense; O, overlapping in the same sense. NR: Location not related to target. ^b^ C: *cis*-acting lncRNA; T: *trans*-acting lncRNA.

**Table 4:** Specific lncRNAs investigated in dimorphic fungi.

Species ^a^	lncRNA	Target	Location ^b^	Functional Role	Mechanism of Action ^c^	Refs.
*Um*	*ncRNA1*	*um12316* *um02150* *um12316*	NR I, DA A	Pathogenesis Pathogenesis Pathogenesis?	T, ND C, ND T, ND	[70]
*Um*	*as-ssm1*	*ssm1*	A	Mitochondrial function	C, duplex RNA formation	[108]
*Um*	*emi1*	Telomerase	NR	Telomerase function	T, Maturation, protein recruiting	[191]
*Ca*	*DINOR*	ND	NR	Genome integrity, Morphological transition	T, protein recruiting?	[192]
*Cn*	*RZE1*	*ZNF2*	I, UA	Morphological transition	C, ND	[193]
*Uv*	*UvlncNAT-MFS*	*UvMFS*	A	Zinc transport	C, duplex RNA formation	[53]

ND: Not determined. ^a^ *Um*, *Ustilago maydis*; *Ca*: *Candida auris*; *Cn*: *Cryptococcus neoformans*; *Uv*: *Ustilaginoidea virens*. ^b^ A, antisense of a coding sequence. I, intergenic: UA, upstream of target in opposite sense; DA, downstream of target in the opposite sense. NR: Location not related to target. ^c^ C: *cis*-acting lncRNA; T: *trans*-acting lncRNA.

**Table 5:** Specific lncRNAs investigated in filamentous fungi.

Species ^a^	lncRNA	Target	Location ^b^	Functional Role ^c^	Mechanism of Action ^d^	Refs.
*Nc*	*qrf*	*frq*	A	Circadian rhythm	C, chromatin remodeling	[198,199,200]
*Tr*	*HAX1*	Xyr1	NR	Cellulase production	T, protein interaction	[112,201]
*Ff/Fo*	*Ff-carP*	*carS*	I, US	Photocarotenogenesis	C, transcription interference	[202,203]
*Fg*	*GzmetE-AS*	*GzmetE*	A	Sexual development	C, sense mRNA degradation	[204]
*Fg*	*anti TRI5* *anti TRI6* *anti TRI11* *RNA5P*	*tri5* *tri6* *tri11*	A A A I, US	Secondary metabolism (DON)	C, ND C, ND C, ND C, transcription interference?	[205]
*Fg*	*lncRsp1*	*Fgsp1*	I, US, O	Sexual development, Secondary metabolism (DON)	ND	[206]
*Fg*	*LncRsn*	*FgSna* *FgPta*	I, DA I, DS	Sexual development, pathogenesis	ND	[207,208]
*Ch*	*cmr1 antisense*	*cmr1*	A	Melanin production	C, ND	[209]
*Mr*	*mrLncRNA1*	ND	ND	Conidia thermotolerance	ND	[87]
*Pi*	*Pinci1-1* *Pinci1-5*	ND	I	Pathogenesis	ND	[210]
*Po*	*G003973*	*MGG_15773*	I, US	Hyphal development	C, ND	[59]
*Rd*	*MSTRG.10396.1*	*RO3G_03340*	ND	Cell wall	ND	[211]
*Vd*	*LNC_01522*	*VDAG_09366* *VDAG_01782* *VDAG_01205*	ND	Pathogenesis	ND	[58]
*Vd*	*LNC_000201*	*VDAG_09366*	ND	Pathogenesis?	ND	[58]
*Vd*	*lncRNA009491* *lncRNA007722* *lncRNA009491*	ND	ND	Pathogenesis	ND	[82]

^a^ *Nc*, *Neurospora crassa*; *Tr*, *Trichoderma reesei*; *Ff/Fo*, *Fusarium fujikuroi/Fusarium oxysporum*; *Fg*, *Fusarium graminearum*; *Ch*, *Cochliobolus heterostrophus*; *Mr*, *Metharhizium robertsii*; *Pi*, *Phytophthora infestans*; *Po*, *Pyricularia oryzae*; *Rd*, *Rhizopus delemar*; *Vd*, *Verticilium dahliae*. ^b^ A, antisense of a coding sequence. I, intergenic: US, upstream of target in the same sense; DS, downstream of target in the same sense; DA, downstream of target in the opposite sense. O, overlapping in the same sense. NR: Location not related to target. ^c^ DON: Deoxynivalenol production. ^d^ C: *cis*-acting lncRNA; T: *trans*-acting lncRNA. ND: Not determined.

## Abbreviations

The following abbreviations are used in this manuscript:

CTD	Carboxy terminal domain
CUT	Cryptic unstable transcript
DON	Deoxynivalenol
DSR	Determinant of selective removal
DUT	Dicer-sensitive unstable transcript
EST	Expressed sequence tag
lncRNA	Long non-coding RNA
lincRNA	Long intergenic non-coding RNA
MUT	Meiotic unannotated transcripts
ncRNA	Non-coding RNA
NMD	Nonsense-mediated decay
NUT	Nrd1-unterminated transcript
snoRNA	small nucleolar RNA
SUT	Stable unannotated transcript
TSS	Transcription start site
XUT	Xrn1-sensitive unstable transcripts XUT
cenRNA	Centromeric RNA
